# Supplementary material for: Optimising implementation strategies of the first scaleup of a primary care psychological intervention for common mental disorders in Sub-Saharan Africa: a mixed methods study protocol for the optimised Friendship Bench (OptFB)
Source: BMJ Open. 2021 Sep 13;11(9):e045481. doi: 10.1136/bmjopen-2020-045481 (PMC8438921; doi:10.1136/bmjopen-2020-045481)
Supplement: Author's manuscript [file bmjopen-2020-045481.draft_revisions.pdf]

BMJ Open

BMJ Open is committed to open peer review. As part of this commitment we make the peer review history of every article we publish publicly available.

When an article is published we post the peer reviewers' comments and the authors' responses online. We also post the versions of the paper that were used during peer review. These are the versions that the peer review comments apply to.

The versions of the paper that follow are the versions that were submitted during the peer review process. They are not the versions of record or the final published versions. They should not be cited or distributed as the published version of this manuscript.

BMJ Open is an open access journal and the full, final, typeset and author-corrected version of record of the manuscript is available on our site with no access controls, subscription charges or pay-per-view fees (http://bmjopen.bmj.com).

If you have any questions on BMJ Open's open peer review process please email info.bmjopen@bmj.com

BMJ Open

# Optimizing implementation strategies of the first scale-up of a primary care psychological intervention for common mental disorders in Sub-Saharan Africa: Study protocol for the Optimized Friendship Bench (OptFB)


| | |
|---|---|
| Journal: | *BMJ Open* |
| Manuscript ID | bmjopen-2020-045481 |
| Article Type: | Protocol |
| Date Submitted by the Author: | 05-Oct-2020 |


| | |
|---|---|
| Complete List of Authors: | Verhey, Ruth ; University of Zimbabwe, Research Support Centre; Friendship Bench<br>Chitiyo, Charmaine; Friendship Bench Zimbabwe<br>Mboweni, Sandra; Friendship Bench Zimbabwe<br>Chiriseri, Ephraim; Friendship Bench Zimbabwe<br>Chibanda, Dixon; Friendship Bench Zimbabwe; University of Zimbabwe, Research Support Centre<br>Healey, Andy; King's College London, IOPPN<br>Wagenaar, Bradley; University of Washington, Department of epidemiology; University of Washington, Department of global health<br>Araya, Ricardo; Centre for Global Mental Health and Primary Care Research, |


| | |
|---|---|
| Keywords: | MENTAL HEALTH, PRIMARY CARE, PUBLIC HEALTH, QUALITATIVE RESEARCH |


SCHOLARONE™
Manuscripts

*I, the Submitting Author has the right to grant and does grant on behalf of all authors of the Work (as defined in the below author licence), an exclusive licence and/or a non-exclusive licence for contributions from authors who are: i) UK Crown employees; ii) where BMJ has agreed a CC-BY licence shall apply, and/or iii) in accordance with the terms applicable for US Federal Government officers or employees acting as part of their official duties; on a worldwide, perpetual, irrevocable, royalty-free basis to BMJ Publishing Group Ltd ("BMJ") its licensees and where the relevant Journal is co-owned by BMJ to the co-owners of the Journal, to publish the Work in this journal and any other BMJ products and to exploit all rights, as set out in our licence.*

*The Submitting Author accepts and understands that any supply made under these terms is made by BMJ to the Submitting Author unless you are acting as an employee on behalf of your employer or a postgraduate student of an affiliated institution which is paying any applicable article publishing charge ("APC") for Open Access articles. Where the Submitting Author wishes to make the Work available on an Open Access basis (and intends to pay the relevant APC), the terms of reuse of such Open Access shall be governed by a Creative Commons licence – details of these licences and which Creative Commons licence will apply to this Work are set out in our licence referred to above.*

*Other than as permitted in any relevant BMJ Author's Self Archiving Policies, I confirm this Work has not been accepted for publication elsewhere, is not being considered for publication elsewhere and does not duplicate material already published. I confirm all authors consent to publication of this Work and authorise the granting of this licence.*

For peer review only

Optimizing implementation strategies of the first scale-up of a primary care psychological intervention for common mental disorders in Sub-Saharan Africa: Study protocol for the Optimized Friendship Bench (OptFB)

Author list:
Ruth Verhey [a,b]
Charmaine Chitiyo [a]
Sandra Mboweni [a]
Ephraim Chiriseri [a]
Dixon Chibanda [a,b,d]
Andy Healy [c]
Bradley H. Wagenaar [e,f]
Ricardo Araya [c]

[a] Friendship Bench Zimbabwe
[b] University of Zimbabwe
[c] King's College, London, UK
[d] London school of hygiene and tropical medicine, LSHTM, UK
[e] Department of Global Health, University of Washington, Settle, WA, USA
[f] Department of Epidemiology, University of Washington, Seattle, WA, USA

Abstract word count: 281
Body word count: 3650

Corresponding author:
Ruth Verhey
Address: 4 Weale Road, Milton Park Harare Zimbabwe
Tel: +263773857376 Email: ruth.verhey@friendshipbench.io


For peer review only - http://bmjopen.bmj.com/site/about/guidelines.xhtml

**Abstract**

Introduction: Common mental disorders (CMDs) are a leading cause of disability globally. CMDs are highly prevalent in Zimbabwe and have been addressed by an evidence-based, task-shifting psychological intervention called the Friendship Bench (FB). The task-shifted FB program guides clients through problem solving therapy. It was scaled-up across 36 implementation sites in Zimbabwe in 2016.

Methods and analysis: This study will employ a mixed-methods framework. It aims to: (1) Use quantitative survey methodologies organized around the RE-AIM evaluation framework to assess the current scale-up of the FB intervention and classify 36 clinics according to levels of performance; (2) Use qualitative focus group discussions and semi-structured interviews organized around the Consolidated Framework for Implementation Research (CFIR) to analyze determinants of implementation success, as well as elucidate heterogeneity in implementation strategies through comparing high- and low-performing clinics; and (3) Use the results from aims 1 and 2 to develop strategies to optimize the Friendship Bench intervention and apply this model in a cluster randomized controlled trial to evaluate potential improvements among low-performing clinics. The trial will be registered with the Pan African Clinical Trial Registry (www.pactr.org). The planned randomized controlled trial for the third research aim will be registered after completing aims one and two because the intervention is dependent on knowledge generated during these phases.

Ethics and dissemination: The research protocol received full authorization from the Medical Research Council of Zimbabwe (MRCZ A/242). It is anticipated that changes in data collection tools and consent forms will take place at all three phases of the study and approval from MRCZ will be sought. All interview partners will be asked for informed consent. The research team will prioritize open access publications to disseminate research results.



For peer review only - http://bmjopen.bmj.com/site/about/guidelines.xhtml

## Strengths and limitations of this study

- Few evidence-based psychological interventions offered at primary health care level have been successfully scaled-up in Sub-Saharan Africa; this study is designed to deliver detailed knowledge about factors that influence the scale-up of a primary care psychological intervention (the Friendship Bench) in an African setting.
- Two widely used implementation science models, RE-AIM and CFIR, will be used to evaluate the implementation of this intervention, which was scaled up in 2016.
- This study focuses on evaluating the scaling up of evidence-based interventions and developing and testing implementation strategies to potentially optimize the routine delivery of the Friendship Bench.
- A limitation is that comprehensive implementation data is only collected three years after the scale up exercise.

Key words: Friendship Bench, Optimization, common mental disorders, CFIR, RE-AIM, Low- and middle-income countries

For peer review only

## 1. Introduction:

In the past 10 years, it has become apparent that mental, neurological and substance use disorders (MNS) are among the leading causes of the global disease burden [1-3]. Research has shown that 4 out of every 10 people in low-and-middle-income countries (LMICs) suffer from mental disorders (de Boer et al. 2008, World Health Organization, 2009a) and evidence-based mental health interventions have become a focus of research and interest [4]. It has been observed that the poor are disproportionately affected by mental disorders [5 6]. Less than 5% of people living in some LMIC receive any adequate treatment for mental health disorders [7 8 9]. Particularly in low- and


For peer review only - http://bmjopen.bmj.com/site/about/guidelines.xhtml

middle-income countries (LMIC) the lack of resources, especially trained mental health professionals, causes sub-optimal detection and management of CMD [10-12]. Worldwide, efforts have been made to create sustainable and affordable mental health interventions in primary care [13-18]. In a recent systematic review, only four studies were detected that had evaluated the implementation of a depression intervention scaled-up in routine care [19]. As it stands, the benefit of these evidence-based interventions is not yet reaching those populations most at need across LMICs.

Zimbabwe, a country in Southern Africa with a population of 13 million has a large treatment gap for MNS. Studies show that over 30% of primary health care (PHC) users need mental health care services for mostly common mental disorders (CMD) and only 5% of these receive appropriate care [20]. Untreated CMD can also lead to worsening of clinical outcomes in chronic conditions such as HIV [21] and negatively affect economic outcomes too [5]. The Friendship Bench (FB) was developed in response to the existing treatment gap for mental health care in Zimbabwe and tested for its efficacy in a cluster randomized controlled trial (RCT) [22].

This task-shifted intervention is delivered by trained and supervised lay health workers (LHWs) who deliver problem solving therapy (PST) [23] on a bench located in primary health care clinics. In 2016, the FB intervention was scaled-up across Harare, Gweru and Chitungwiza and surrounding peri-urban communities in collaboration with the respective City Health departments [24]. The FB program was established in 72 City Health PHC clinics that are established in 36 sites (different clinic types can be found in the same site). This scaling-up exercise involved the training of more than 300 LHWs in the 3 cities in Zimbabwe [24]. Maintenance funding for FB activities is provided by the City Health department.

All lay health workers (LHWs) working for the FB PHC clinics in Harare, Gweru and Chitungwiza received the standard manualized training and supervision. While existing scientific evidence has shown that under ideal randomized trial conditions the FB intervention leads to clinically-significant reductions in symptoms, little implementation research has been carried out regarding the performance of Friendship Bench under routine conditions as the model is being further scaled-up across Zimbabwe.



For peer review only - http://bmjopen.bmj.com/site/about/guidelines.xhtml

This study will be of interest to implementation scientists, policymakers, and researchers working to scale-up primary care psychological interventions in low- and middle-income countries (LMICs) globally. Results from this study have the potential to inform future scale-up and maintenance of task-shared psychological interventions into routine Ministry of Health primary care settings.

## 2. Overall Study Goal

This research uses a mixed-methods study design and widely-used implementation frameworks to systematically analyze the performance of clinics, determinants of this performance, including implementation strategies that might differentiate high- versus low-performing clinics, and develop and test an enhanced implementation strategy to improve the performance of clinics in three cities in Zimbabwe. The study is designed to be conducted in three phases with corresponding aims.

Firstly (aim 1), we plan to examine how the FB is performing under real-world implementation conditions and classify existing clinics with FB into high- versus low-performing sites using differences in RE-AIM outcomes [25 26].

Secondly (aim 2), we will analyse the determinants of heterogeneity in the results of phase 1 comparing high- versus low-performing clinics, mainly using the CFIR framework [27] and rigorously documenting changes to the original FB protocol and current implementation strategies in use.

Thirdly (aim 3), we will develop and test an optimized package of FB implementation strategies based on the results of phase 2 and measure the improvement among low performing clinics using RE-AIM outcomes.

## 3. Study setting:

The study will be conducted in primary health care clinics (PHC) in Harare, Gweru, and Chitungwiza.

Most of the clinics in the 3 cities are located in comparable areas which are characterized by high population density and informal income generating activities often occurring in the vicinity of the clinics. Depending on their size, PHC clinics serve between 20,000-80,000 people from the most socio-economically disadvantaged sectors of the population. Clinics are differentiated into poly, satellite, and family health service clinics according to the size of the clinic and the range of services offered.


For peer review only - http://bmjopen.bmj.com/site/about/guidelines.xhtml

The most comprehensive services are offered in a Polyclinic such as pre-, post- and perinatal care, opportunistic infections (for example TB treatment), and specialized NGO-based programs (HIV testing and management, male circumcision, communicable disease awareness). Satellite and Family health clinics (FHS) offer less services. Medical doctors are not permanently present but hold clinics on specific days in poly clinics. This influences the clinic user population's composition on these particular days (for example HIV clinic day).

Clinics in Harare, Chitungwiza and Gweru are grouped and located in the same geographical facility and these are counted as one Friendship Bench implementation site. Data will be collected in 36 implementation sites (n=28 in Harare; n=4 in Gweru; n=4 in Chitungwiza). Of these 26 Poly clinics, six are FHS and four satellite clinics (see figure 1).

Figure 1: Clinic type distribution for Harare, Gweru and Chitungwiza

Depending on their size and catchment area, FB implementation sites have between one (1) and fourteen (14) LHWs who deliver the FB intervention on benches in the clinic premises during clinic opening times. Clinic users are informed about the about FB services and mental health through group or individual talks in the clinic's waiting areas. Community members are also directly in contact with LHWs during outreach activities in the community.

## 4. Methods

This study proposes a rigorous analysis of the multiple interconnecting factors using two internationally recognized implementation research methods – the RE-AIM model [26] and the CFIR [27] which will be described in more detail below. Both conceptual frameworks have been used widely in implementation research for health care delivery in order to deepen the understanding and evaluation of interventions such as the Friendship Bench. The study has three research aims which are linked contextually to each other and are described in detail below.



For peer review only - http://bmjopen.bmj.com/site/about/guidelines.xhtml

### 4.1 Methods Aim 1

A thorough analysis of the existing routine health information system data collected by the Harare, Gweru and Chitungwiza City Health authorities will be carried out to learn about the Friendship Bench activities at individual clinic level. This data consists of user numbers, age, gender, HIV status, clients' screening tool scores, and number of sessions.

We will use the RE-AIM evaluation framework to evaluate the current implementation performance of the FB intervention after three years of implementation experience. Routinely collected data will be used to assess the FB intervention's real-world and pragmatic performance: Reach, Effectiveness, Adoption, Implementation, and Maintenance. The research team which consists of experienced global mental health researchers and clinicians will develop indicators for each of the RE-AIM domains based on expert consensus and availability of data. These indicators will then be used to design a questionnaire to guide the RE-AIM related data collection. Each indicator will comprise a numerator and a denominator populated with data collected from the clinic records and the planned observations.

The data on the FB implementation will be analyzed for each of the 36 participating clinics. Routinely collected data includes clinical registries for both nurses and LHWs and data from the FB Register (commonly known as the "green book") where the LHWs record beneficiary information.

In addition, LHWs will be observed during all aspects of their work, including giving health talks, interacting with clients, and delivering the FB intervention. We will observe and record whether all FB related tools such as questionnaires and intervention tools are used.

In order to collect additional necessary data for AIM 1, key respondents will be interviewed using a questionnaire that will be developed by the research team.

We plan to interview at least 2 LHWs per clinic and in clinics with more than 2 LHWs, we will interview 50% of the present LHWs by randomly selecting them. Papers with their names will be put in a container from which a RA will pull out the appropriate number in the LHWs' presence. We will always interview the supervisor LHW of each clinic if this position is taken in a particular clinic. We will also interview the nurse in


For peer review only - http://bmjopen.bmj.com/site/about/guidelines.xhtml

charge in every clinic and the associated district health promoting officers (DHPOs) (n=10). Data will be collected from June to September 2019 in all participating sites.

The data collection will be carried out by two research coordinators who will lead two teams of four trained and supervised research assistants (RAs). The teams will visit each clinic for two days. The clinics will be sensitized about the FB team visit a week prior. The research assistants will be trained to interview, to observe and record the FB related activities in the clinic and how to enter the data digitally using tablet computers. They will be trained on data checking, cleaning and uploading.

Furthermore, we are planning to audio-record FB sessions with consenting clients (two per site, n=72). The recordings will be translated, transcribed and rated according to the Friendship Bench fidelity checklist.
The FB fidelity checklist assesses for communication skills of the counselor, the level of psychoeducation that is done, and the adherence to the problem-solving therapy steps that the FB counselor is trained to deliver (see Supplementary Appendix A for full fidelity checklist which was developed for the RCT [22]). The assessments of audio recordings will be done by trained FB research team members who will prepare an audio-recorder which will be left with the FB counselor after a client has given consent. The audio recording device will be retrieved by the research assistant when the LHW has indicated that the session is done.
In the event that no clients come to the clinic on both days that the FB team visits the site or no client consents to have their session audio-recorded, this will be entered as missing. Due to logistic and financial constraints a repeat visit to a particular clinic will not be possible.
All respondents will be asked to answer the questions with regards to FB activities in the past month. According to their position with regards to FB activities, questions might be formulated slightly differently.

The questionnaires will be administered using tablet computers (Lenovo), all observational data will be entered digitally after their correctness has been ascertained by asking interviewees to show evidence as applicable. Questionnaires and observation guides are programmed into the tablets using Kobotoolbox



For peer review only - http://bmjopen.bmj.com/site/about/guidelines.xhtml

(https://www.kobotoolbox.org) which is a data collection tool. Collected data will be cleaned and uploaded daily to a password secured server.

The research team will also observe FB specific activities such as health and ‘mobilization’ talks that are given by the clinic staff including the LHWs whilst patients are waiting to be seen.

A stakeholder meeting will be held once Aim 1 data is completed and the data is analyzed. At this meeting, the research team will present the results from Aim 1 and discuss potential reasons why we might see the differences in implementation across sites with stakeholders. This meeting will be attended by all relevant clinic staff, health authority officers as well as clients. Information from stakeholders will be used to select and prioritize CFIR constructs to include in qualitative interview guides for Aim 2.

**4.1.1 Data Analysis Aim 1**

The goal of Aim 1 is to classify the 36 FB implementation sites on their performance based on the RE-AIM outcomes. Our methods will follow similar classification efforts previously published [28]. Clinics will be first ranked according to their performance within each individual measure. Clinics score on all indicators within one construct (for example reach) will be averaged. For each of the RE-AIM constructs, every clinic will thus have an averaged ranking.

These domain-based rankings will be averaged per clinic rankings giving an overall ranking by calculating simple means of all domain rankings. In case of same outcomes for clinics, we will treat these particular clinics as being on the same rank. This will give us a final composite rank for each clinic which will be used to determine the 10 highest and 10 lowest performing clinics that will be qualitatively assessed in Aim 2.

**4.2 Methods Aim 2**

With the aim to understand the determinants of implementation success, as well as differences in implementation strategies employed, Aim 2 will utilize focus-group discussions organized around the Consolidated Framework for Implementation Research (CFIR) [29-31]. Through these qualitative methods, we aim to gain a deeper



For peer review only - http://bmjopen.bmj.com/site/about/guidelines.xhtml

understanding of the factors that contribute to the successful implementation comparing high- with low-performing clinics. The CFIR framework focuses on an overview of potential multi-level determinants of health care delivery. It was designed to help understand integrated implementation determinants across multiple levels (clients; implementers; organizations; contexts; processes).

For the present study, we will focus on determinants of implementation success, taking lessons from both high- and low-performing clinics to inform the development of an improved package of implementation strategies targeting identified barriers.

Focus group discussions (FDGs) with key informants (LHWs, nurses, DHPOs, clients) of the 10 high and 10 low performing clinics will be carried out by trained qualitative researchers. The FB specific interview guides for these group discussions and interviews will be developed by the study team in a sequence of internal project meetings using the online technical support website www.cfirguide.org. The results of Aim 1 will guide us in designing the interview guides for the focus group discussions.

The outcome of the stakeholders meeting in which we present the results of Aim 1 will also give us insight on the importance of constructs which we will take into account when designing the CFIR interview guides.

Interview guides will be translated into the local language Shona and all group discussions will be audio-recorded, transcribed and translated to English. All discussions will be held in the local language.

The FGD participants will be selected from all 10 low and high performing clinics, respectively. We will interview LHWs, nurses, DHPOs in their role as implementers as well as clients as recipients of the intervention. Focus group discussions will take place in clinics or, if not possible, in the Friendship Bench office in Harare.

**4.2 1 Data Analysis Aim 2**

CFIR analyses will follow the original Damschroder methodology previously published [30]. Briefly, two independent local Zimbabwean reviewers will code each FGD transcript according to the selected CFIR constructs. Differences will be discussed and revised until final codes are agreed on. Facility-level case memos will be organized by the relevant CFIR construct, using each new transcript to confirm and refine statements until all transcripts are coded. This process will be closely supported by the whole


For peer review only - http://bmjopen.bmj.com/site/about/guidelines.xhtml

research team. Each clinic will have two case memos, one for LHWs and other implementers and one for clients.

Using case memos and supporting transcripts, the same two coders will independently rate CFIR constructs on valence (X (mixed); 0 (neutral); + (construct has a positive effect on implementation) or – (construct has a negative influence on implementation). Once drafted, the entire research team will meet and use a deliberated consensus to finalize memos, constructs, and valence. These data will be mapped on a matrix template with the goal of identifying constructs that differ between facilities with high and low performance to identify factors relevant for the success of the implementation. Analyses will progress with visual inspection of patterns in constructs and valence by high versus low performing clinics, as well as examining median and mean valence by high versus low performing clinics. Once distinguishing constructs are identified, the team will re-review case memos and coded transcripts to gather more information on constructs.

### 4.3 Aim 3

In Aim 3, we will develop a package of optimized Friendship Bench (OptFB) implementation strategies matched to key barriers identified in the previous phases of this study. Using CFIR data on barriers / facilitators to high-quality FB implementation, we will use the CFIR-ERIC matching tool to examine and select implementation strategies to address key CFIR constructs discriminating between high and low performing clinics in Aim 1 (https://cfirguide.org/choosing-strategies/) [32 33]. Once a preliminary list is developed by our team, the CFIR-Expert Recommendation for Implementation Change (ERIC) matching tool [32] will be used to prioritize those strategies that are found to be most likely to address CFIR barriers in low-performing clinics [33 34].

We will engage in a participatory stakeholder Delphi rating exercise to select specific strategies. This will be followed by the research team specifying and tailoring the strategies for the Zimbabwean context by including the additional information gained from the stakeholders. Aspects of feasibility, affordability and effectiveness will guide this process in order for the package to be meaningful and effective [35]. Strategies currently in use by high performing clinics will be also considered for the optimized Friendship Bench (OptFB) implementation strategies.


For peer review only - http://bmjopen.bmj.com/site/about/guidelines.xhtml

This OptFB package or intervention of improved strategies will be tested in low-performing clinics. Ongoing RE-AIM data is being collected on a monthly basis in each clinic. Using these data on RE-AIM outcomes, we will re-classify clinics using a similar process as in Aim 1. We will then identify the 18 lowest performing clinics and randomly select 12 clinics to deliver the OptFB and 6 to act as control clinics over a period of 6 months. The primary outcome will be a composite measure of RE-AIM indicators (Reach, Effectiveness, Adoption and Implementation and Maintenance) estimated at 6-month after the commencement of the implementation of the OptFB intervention. We will estimate changes in this composite measure of implementation before and at 6 months after starting the delivery of OptFB in all clinics. We will compare the difference in means or proportions between the clinics receiving the OptFB and the control clinics using the routinely collected data. Secondary outcomes will examine performance of each of the RE-AIM outcomes separately and clinical effectiveness results at individual level. The latter will be based on individual scores to SSQ on a minimum of 20 random individuals per clinic during the 6-month period.

No sample size calculation has been estimated since there are no previous studies on which to estimate an effect size, the number of clinics is small, and the main outcomes are averaged data representing clusters. Nonetheless, we expect to see larger improvements in the RE-AIM composite index score in the clinics receiving OptFB compared to the control clinics over the 6 months. As a secondary outcome measure, clinical effectiveness will be assessed based on changes on SSQ scores from baseline to 6 months for a sample of 360 individuals (18 clinics with 20 individuals each), but we do not expect this sample would have enough power to detect small differences in effectiveness across the two group of clinics. Thus, comparisons on clinical effectiveness must be considered purely descriptive and exploratory and interpreted with caution. In any case, the main outcomes of interest in this study are implementation outcomes subsumed under the domains included in the RE-AIM framework.

### 4.3.1 Data analysis Aim 3

We will use a difference-in-differences analysis comparing the groups over time. Means or proportions on outcome data will be compared across groups using descriptive statistics. Regression models will be used to estimate the effect of the intervention on the main outcomes. General estimating equations with robust

For peer review only

standard errors will be used to control for clustering. Potential confounders will be determined a priori and included in the regression models. Standard errors, confidence intervals, and p-values will be obtained. A similar secondary analysis will be conducted with the secondary outcome measures.

### 4.4 Health economic analysis

Site-level data will be collected on fidelity to the OptFB implementation strategies, along with activities and resource inputs required to deliver improvement strategies and OptFB delivery costs. Economic modelling will be used to combine this information with data and evidence on clinical impact and implementation effectiveness to evaluate the cost-effectiveness of the OptFB program [36].

We will also revisit clinics and re-engage with stakeholders into FGD to explore level of change in the identified CFIR domains in the intervention arm clinics.

After completion of the trial, the strategy will also be implemented in the control arm clinics to increase the overall performance in all of participating lower performing clinics.

## 5. Discussion

This study will contribute to the knowledge about scaling up of an evidence-based task-shifted intervention in a LMIC. This is a unique opportunity to analyze the Friendship Bench in a real-world setting. As mentioned above, not many interventions have been scaled up from LMICs and therefore there is a dearth of information on how implementation strategies can be used in order to ensure a strong scaling up. With this study we hope to learn which barriers and enablers are at play in the FB scale up process. This is particularly important for us as we are expanding the FB services throughout Zimbabwe and beyond to meet the population's needs for accessible and acceptable mental health care. This effort has to be undertaken with the aim of having high fidelity to the program while considering contextual aspects. Using implementation science principles will help us to give theoretical justification and describe specifications for application for those implementation strategies that we will devise after having gone through the different stages of this research process. Evidence-based, clear and applicable guidelines of


For peer review only - http://bmjopen.bmj.com/site/about/guidelines.xhtml

how to implement our evidence-based intervention in primary health care settings will be created and can then subsequently be used to ensure a strong implementation of FB.

## References


1. Vigo D, Thornicroft G, Atun R. Estimating the true global burden of mental illness. *Lancet Psychiatry* 2016;3(2):171-8. doi: 10.1016/S2215-0366(15)00505-2 [published Online First: 2016/02/07]
2. Kessler RC, Aguilar-Gaxiola S, Alonso J, et al. The global burden of mental disorders: an update from the WHO World Mental Health (WMH) surveys. *Epidemiol Psichiatr Soc* 2009;18(1):23-33. doi: 10.1017/s1121189x00001421 [published Online First: 2009/04/22]
3. Whiteford HA, Ferrari AJ, Degenhardt L, et al. The global burden of mental, neurological and substance use disorders: an analysis from the Global Burden of Disease Study 2010. *PLoS One* 2015;10(2):e0116820. doi: 10.1371/journal.pone.0116820 [published Online First: 2015/02/07]
4. Steel Z, Marnane C, Iranpour C, et al. The global prevalence of common mental disorders: a systematic review and meta-analysis 1980–2013. *International journal of epidemiology* 2014:dyu038.
5. Lund C, De Silva M, Plagerson S, et al. Poverty and mental disorders: breaking the cycle in low-income and middle-income countries. *The Lancet* 2011;378(9801):1502-14.
6. Evans-Lacko S, Aguilar-Gaxiola S, Al-Hamzawi A, et al. Socio-economic variations in the mental health treatment gap for people with anxiety, mood, and substance use disorders: results from the WHO World Mental Health (WMH) surveys. *Psychological medicine* 2018;48(9):1560-71. doi: 10.1017/S0033291717003336 [published Online First: 2017/11/28]
7. Wang PS, Aguilar-Gaxiola S, Alonso J, et al. Use of mental health services for anxiety, mood, and substance disorders in 17 countries in the WHO world mental health surveys. *The Lancet* 2007;370(9590):841-50.
8. Mojtabai R, Olfson M, Sampson NA, et al. Barriers to mental health treatment: results from the National Comorbidity Survey Replication. *Psychological medicine* 2011;41(8):1751-61. doi: 10.1017/S0033291710002291 [published Online First: 2010/12/08]


For peer review only

9. Kohn R, Saxena S, Levav I, et al. The treatment gap in mental health care. *Bull World Health Organ* 2004;82(11):858-66. doi: /S0042-96862004001100011 [published Online First: 2005/01/11]
10. Prince M, Patel V, Saxena S, et al. No health without mental health. *The lancet* 2007;370(9590):859-77.
11. Alonso J, Liu Z, Evans-Lacko S, et al. Treatment gap for anxiety disorders is global: Results of the World Mental Health Surveys in 21 countries. *Depress Anxiety* 2018;35(3):195-208. doi: 10.1002/da.22711 [published Online First: 2018/01/23]
12. Wang PS, Angermeyer M, Borges G, et al. Delay and failure in treatment seeking after first onset of mental disorders in the World Health Organization's World Mental Health Survey Initiative. *World Psychiatry* 2007;6(3):177-85. [published Online First: 2008/01/12]
13. Araya R, Rojas G, Fritsch R, et al. Treating depression in primary care in low-income women in Santiago, Chile: a randomised controlled trial. *Lancet* 2003;361(9362):995-1000. doi: 10.1016/S0140-6736(03)12825-5
14. Patel V, Thornicroft G. Packages of care for mental, neurological, and substance use disorders in low-and middle-income countries: PLoS Medicine Series. *PLoS medicine* 2009;6(10):e1000160.
15. Chatterjee S, Chowdhary N, Pednekar S, et al. Integrating evidence-based treatments for common mental disorders in routine primary care: feasibility and acceptability of the MANAS intervention in Goa, India. *World Psychiatry* 2008;7(1):39-46.
16. Rahman A, Malik A, Sikander S, et al. Cognitive behaviour therapy-based intervention by community health workers for mothers with depression and their infants in rural Pakistan: a cluster-randomised controlled trial. *The Lancet* 2008;372(9642):902-09.
17. Bolton P, Bass J, Neugebauer R, et al. Group interpersonal psychotherapy for depression in rural Uganda: a randomized controlled trial. *Jama* 2003;289(23):3117-24.
18. Lund C, Alem A, Schneider M, et al. Generating evidence to narrow the treatment gap for mental disorders in sub-Saharan Africa: rationale, overview and methods of AFFIRM. *Epidemiol Psychiatr Sci* 2015;24(3):233-40. doi: 10.1017/S2045796015000281 [published Online First: 2015/04/03]
19. Wagenaar BH, Hammett, W.H., Jackson, C., Atkins, D.L., Belus, J.M. and Kemp, C.G. Implementation outcomes and strategies for depression interventions in low-and middle-income countries: a systematic review. . *Global Mental Health* 2020;7
20. Chibanda D, Mesu P, Kajawu L, et al. Problem-solving therapy for depression and common mental disorders in Zimbabwe: piloting a task-shifting primary mental health care intervention in a population with a high prevalence of people living with HIV. *BMC public health* 2011;11:828. doi: 10.1186/1471-2458-11-828 [published Online First: 2011/10/28]
21. Antelman G, Kaaya S, Wei R, et al. Depressive symptoms increase risk of HIV disease progression and mortality among women in Tanzania. *Journal of acquired immune deficiency syndromes* 2007;44(4):470-7. doi: 10.1097/QAI.0b013e31802f1318
22. Chibanda D, Weiss HA, Verhey R, et al. Effect of a Primary Care-Based Psychological Intervention on Symptoms of Common Mental Disorders in Zimbabwe: A Randomized Clinical Trial. *JAMA* 2016;316(24):2618-26. doi: 10.1001/jama.2016.19102 [published Online First: 2016/12/28]
23. Chibanda D, Bowers T, Verhey R, et al. The Friendship Bench programme: a cluster randomised controlled trial of a brief psychological intervention for common mental

For peer review only

disorders delivered by lay health workers in Zimbabwe. *Int J Ment Health Syst* 2015;9:21. doi: 10.1186/s13033-015-0013-y [published Online First: 2015/01/01]
24. Chibanda D, Verhey R, Munetsi E, et al. Scaling up interventions for depression in sub-Saharan Africa: lessons from Zimbabwe. *Glob Ment Health (Camb)* 2016;3:e13. doi: 10.1017/gmh.2016.8 [published Online First: 2016/04/11]
25. Glasgow RE, Vogt TM, Boles SM. Evaluating the public health impact of health promotion interventions: the RE-AIM framework. *Am J Public Health* 1999;89(9):1322-27.
26. Glasgow RE, Vogt TM, Boles SM. Evaluating the public health impact of health promotion interventions: the RE-AIM framework. *American journal of public health* 1999;89(9):1322-7. doi: 10.2105/ajph.89.9.1322 [published Online First: 1999/09/04]
27. Damschroder LJ, Aron DC, Keith RE, et al. Fostering implementation of health services research findings into practice: a consolidated framework for advancing implementation science. *Implement Sci* 2009;4:50. doi: 10.1186/1748-5908-4-50 [published Online First: 2009/08/12]
28. Farris RP, Will JC, Khavjou O, et al. Beyond effectiveness: evaluating the public health impact of the WISEWOMAN program. *American journal of public health* 2007;97(4):641-47.
29. Breimaier HE, Heckemann B, Halfens RJ, et al. The Consolidated Framework for Implementation Research (CFIR): a useful theoretical framework for guiding and evaluating a guideline implementation process in a hospital-based nursing practice. *BMC Nurs* 2015;14:43. doi: 10.1186/s12912-015-0088-4 [published Online First: 2015/08/14]
30. Damschroder LJ, Lowery JC. Evaluation of a large-scale weight management program using the consolidated framework for implementation research (CFIR). *Implement Sci* 2013;8:51. doi: 10.1186/1748-5908-8-51 [published Online First: 2013/05/15]
31. Damschroder LJ, Aron DC, Keith RE, et al. Fostering implementation of health services research findings into practice: a consolidated framework for advancing implementation science. *Implementation science* 2009;4(1):50.
32. Powell BJ, Waltz TJ, Chinman MJ, et al. A refined compilation of implementation strategies: results from the Expert Recommendations for Implementing Change (ERIC) project. *Implement Sci* 2015;10:21. doi: 10.1186/s13012-015-0209-1 [published Online First: 2015/04/19]
33. Waltz TJ, Powell BJ, Fernandez ME, et al. Choosing implementation strategies to address contextual barriers: diversity in recommendations and future directions. *Implement Sci* 2019;14(1):42. doi: 10.1186/s13012-019-0892-4 [published Online First: 2019/05/01]
34. Godbee K, Gunn J, Lautenschlager NT, et al. Refined conceptual model for implementing dementia risk reduction: incorporating perspectives from Australian general practice. *Aust J Prim Health* 2020;26(3):247-55. doi: 10.1071/PY19249 [published Online First: 2020/05/28]
35. Cooper LA, Hill MN, Powe NR. Designing and evaluating interventions to eliminate racial and ethnic disparities in health care. *J Gen Intern Med* 2002;17(6):477-86. doi: 10.1046/j.1525-1497.2002.10633.x [published Online First: 2002/07/23]
36. Briggs A, Sculpher, M., & Claxton, K. . Decision modelling for health economic evaluation. Oxford: Oup Oxford 2006.



For peer review only - http://bmjopen.bmj.com/site/about/guidelines.xhtml

## Footnotes

### Contributors

RA designed the study and received the grant. RA is the overall PI. RV and DC are leading the study locally. CC will have oversight as project coordinator together with SM as her assistant over the data collection in all phases. BW is leading on the implantation science aspects. EC is conducting data cleaning and assisting with analysis. AH is leading on the health economics analysis. All authors will contribute to the development of questionnaires, interview guides and the strategies for the intervention. RV wrote the first version of the protocol paper. All authors contributed by critically reviewing all further drafts and approving of the final paper.

### Funding

This work was supported by Global Alliance for Chronic Diseases (GACD) through the Medical Research Council Grant number: MRC UKRI MR/S004270/1.

**Competing interests** None declared.

**Patient and public involvement** Patients and/or the public will be involved in the stakeholder meetings, they were not involved in the design, nor will they be involved in the study conduct, or reporting, or dissemination plans of this research project.

**Patient consent for publication** Not required.



For peer review only - http://bmjopen.bmj.com/site/about/guidelines.xhtml

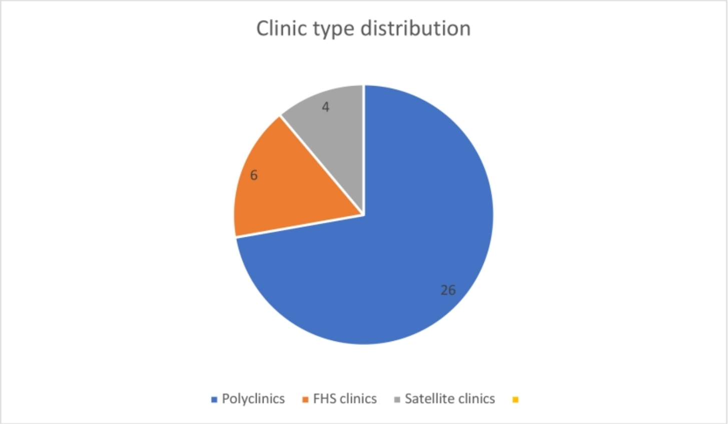


Figure 1: Clinic type distribution for Harare, Gweru and Chitungwiza

## Table 1. Checklist for audio-recorded sessions

## LHW FIDELITY CHECKLIST

**LHW Name________________________ Client's Name________________________**
**Date / / Site Code** ☐☐

| | (Tick where appropriate) | YES | NO |
|---|---|---|---|
| 1. | LHW introduced self to client and asked client to introduce self | ☐ | ☐ |
| 2. | Psycho-education done properly | | |
| | a. Linked HIV to kufungisisa | ☐ | ☐ |
| | b. Adherence | ☐ | ☐ |
| | c. Diet advise done | ☐ | ☐ |
| 3. | Problems presented by client | ☐ | ☐ |
| 4. | LHW listening and acknowledging | ☐ | ☐ |
| 5. | LHW gives summary of problems | ☐ | ☐ |
| 6. | Client selected problem, not LHW | ☐ | ☐ |
| 7. | LHW and client discuss problem identified by client | ☐ | ☐ |
| 8. | Client identifies solutions to the problem identified | ☐ | ☐ |
| 9. | LHW and client identify task for client to work with | ☐ | ☐ |
| 10. | Session closure and next correct review date | ☐ | ☐ |
| | ***Total*** | ____/10 | ___/10 |

For peer review only

# Reporting checklist for protocol of a clinical trial.

Based on the SPIRIT guidelines.

## Instructions to authors

Complete this checklist by entering the page numbers from your manuscript where readers will find each of the items listed below.

Your article may not currently address all the items on the checklist. Please modify your text to include the missing information. If you are certain that an item does not apply, please write "n/a" and provide a short explanation.

Upload your completed checklist as an extra file when you submit to a journal.

In your methods section, say that you used the SPIRITreporting guidelines, and cite them as:

Chan A-W, Tetzlaff JM, Gøtzsche PC, Altman DG, Mann H, Berlin J, Dickersin K, Hróbjartsson A, Schulz KF, Parulekar WR, Krleža-Jerić K, Laupacis A, Moher D. SPIRIT 2013 Explanation and Elaboration: Guidance for protocols of clinical trials. BMJ. 2013;346:e7586

| | | Reporting Item | Page Number |
|---|---|---|---|
| **Administrative information** | | | |
| Title | #1 | Descriptive title identifying the study design, population, interventions, and, if applicable, trial acronym | 1 |
| Trial registration | #2a | Trial identifier and registry name. If not yet registered, name of intended registry | n/a |
| Trial registration: data set | #2b | All items from the World Health Organization Trial Registration Data Set | n/a |
| Protocol version | #3 | Date and version identifier | |
| Funding | #4 | Sources and types of financial, material, and other support | 2 |
| Roles and responsibilities: contributorship | #5a | Names, affiliations, and roles of protocol contributors | 17 |

For peer review only

| | | | |
|---|---|---|---|
| Roles and responsibilities: sponsor contact information | #5b | Name and contact information for the trial sponsor | n/a |
| Roles and responsibilities: sponsor and funder | #5c | Role of study sponsor and funders, if any, in study design; collection, management, analysis, and interpretation of data; writing of the report; and the decision to submit the report for publication, including whether they will have ultimate authority over any of these activities | n/a |
| Roles and responsibilities: committees | #5d | Composition, roles, and responsibilities of the coordinating centre, steering committee, endpoint adjudication committee, data management team, and other individuals or groups overseeing the trial, if applicable (see Item 21a for data monitoring committee) | n/a |
| **Introduction** | | | |
| Background and rationale | #6a | Description of research question and justification for undertaking the trial, including summary of relevant studies (published and unpublished) examining benefits and harms for each intervention | 3 |
| Background and rationale: choice of comparators | #6b | Explanation for choice of comparators | n/a |
| Objectives | #7 | Specific objectives or hypotheses | 4 |
| Trial design | #8 | Description of trial design including type of trial (eg, parallel group, crossover, factorial, single group), allocation ratio, and framework (eg, superiority, equivalence, non-inferiority, exploratory) | 4 |
| **Methods: Participants, interventions, and outcomes** | | | |
| Study setting | #9 | Description of study settings (eg, community clinic, academic hospital) and list of countries where data will be collected. Reference to where list of study sites can be obtained | 5 |

For peer review only

| | | | |
|---|---|---|---|
| Eligibility criteria | #10 | Inclusion and exclusion criteria for participants. If applicable, eligibility criteria for study centres and individuals who will perform the interventions (eg, surgeons, psychotherapists) | 7 |
| Interventions: description | #11a | Interventions for each group with sufficient detail to allow replication, including how and when they will be administered | 7-11 |
| Interventions: modifications | #11b | Criteria for discontinuing or modifying allocated interventions for a given trial participant (eg, drug dose change in response to harms, participant request, or improving / worsening disease) | n/a |
| Interventions: adherance | #11c | Strategies to improve adherence to intervention protocols, and any procedures for monitoring adherence (eg, drug tablet return; laboratory tests) | n/a |
| Interventions: concomitant care | #11d | Relevant concomitant care and interventions that are permitted or prohibited during the trial | n/a |
| Outcomes | #12 | Primary, secondary, and other outcomes, including the specific measurement variable (eg, systolic blood pressure), analysis metric (eg, change from baseline, final value, time to event), method of aggregation (eg, median, proportion), and time point for each outcome. Explanation of the clinical relevance of chosen efficacy and harm outcomes is strongly recommended | 9-10 |
| Participant timeline | #13 | Time schedule of enrolment, interventions (including any run-ins and washouts), assessments, and visits for participants. A schematic diagram is highly recommended (see Figure) | n/a |
| Sample size | #14 | Estimated number of participants needed to achieve study objectives and how it was determined, including clinical and statistical assumptions supporting any sample size calculations | n/a |
| Recruitment | #15 | Strategies for achieving adequate participant enrolment to reach target sample size | n/a |
| **Methods: Assignment of interventions (for controlled trials)** | | | |
| Allocation: sequence generation | #16a | Method of generating the allocation sequence (eg, computer-generated random numbers), and list of any factors for stratification. To reduce predictability of a random sequence, details of any planned restriction (eg, blocking) should be | n/a |

For peer review only

| | | | |
|---|---|---|---|
| | | provided in a separate document that is unavailable to those who enrol participants or assign interventions | |
| Allocation concealment mechanism | #16b | Mechanism of implementing the allocation sequence (eg, central telephone; sequentially numbered, opaque, sealed envelopes), describing any steps to conceal the sequence until interventions are assigned | n/a |
| Allocation: implementation | #16c | Who will generate the allocation sequence, who will enrol participants, and who will assign participants to interventions | n/a |
| Blinding (masking) | #17a | Who will be blinded after assignment to interventions (eg, trial participants, care providers, outcome assessors, data analysts), and how | n/a |
| Blinding (masking): emergency unblinding | #17b | If blinded, circumstances under which unblinding is permissible, and procedure for revealing a participant's allocated intervention during the trial | n/a |
| **Methods: Data collection, management, and analysis** | | | |
| Data collection plan | #18a | Plans for assessment and collection of outcome, baseline, and other trial data, including any related processes to promote data quality (eg, duplicate measurements, training of assessors) and a description of study instruments (eg, questionnaires, laboratory tests) along with their reliability and validity, if known. Reference to where data collection forms can be found, if not in the protocol | 7-10 |
| Data collection plan: retention | #18b | Plans to promote participant retention and complete follow-up, including list of any outcome data to be collected for participants who discontinue or deviate from intervention protocols | n/a |
| Data management | #19 | Plans for data entry, coding, security, and storage, including any related processes to promote data quality (eg, double data entry; range checks for data values). Reference to where details of data management procedures can be found, if not in the protocol | 9-10 |
| Statistics: outcomes | #20a | Statistical methods for analysing primary and secondary outcomes. Reference to where other details of the statistical analysis plan can be found, if not in the protocol | 9-10, 12 |

For peer review only

| | | | |
|---|---|---|---|
| Statistics: additional analyses | #20b | Methods for any additional analyses (eg, subgroup and adjusted analyses) | n/a |
| Statistics: analysis population and missing data | #20c | Definition of analysis population relating to protocol non-adherence (eg, as randomised analysis), and any statistical methods to handle missing data (eg, multiple imputation) | n/a |
| **Methods: Monitoring** | | | |
| Data monitoring: formal committee | #21a | Composition of data monitoring committee (DMC); summary of its role and reporting structure; statement of whether it is independent from the sponsor and competing interests; and reference to where further details about its charter can be found, if not in the protocol. Alternatively, an explanation of why a DMC is not needed | n/a |
| Data monitoring: interim analysis | #21b | Description of any interim analyses and stopping guidelines, including who will have access to these interim results and make the final decision to terminate the trial | n/a |
| Harms | #22 | Plans for collecting, assessing, reporting, and managing solicited and spontaneously reported adverse events and other unintended effects of trial interventions or trial conduct | n/a |
| Auditing | #23 | Frequency and procedures for auditing trial conduct, if any, and whether the process will be independent from investigators and the sponsor | n/a |
| **Ethics and dissemination** | | | |
| Research ethics approval | #24 | Plans for seeking research ethics committee / institutional review board (REC / IRB) approval | 2 |
| Protocol amendments | #25 | Plans for communicating important protocol modifications (eg, changes to eligibility criteria, outcomes, analyses) to relevant parties (eg, investigators, REC / IRBs, trial participants, trial registries, journals, regulators) | 2 |
| Consent or assent | #26a | Who will obtain informed consent or assent from potential trial participants or authorised surrogates, and how (see Item 32) | 8 |

For peer review only

| | | | |
|---|---|---|---|
| Consent or assent: ancillary studies | #26b | Additional consent provisions for collection and use of participant data and biological specimens in ancillary studies, if applicable | n/a |
| Confidentiality | #27 | How personal information about potential and enrolled participants will be collected, shared, and maintained in order to protect confidentiality before, during, and after the trial | n/a |
| Declaration of interests | #28 | Financial and other competing interests for principal investigators for the overall trial and each study site | 17 |
| Data access | #29 | Statement of who will have access to the final trial dataset, and disclosure of contractual agreements that limit such access for investigators | |
| Ancillary and post trial care | #30 | Provisions, if any, for ancillary and post-trial care, and for compensation to those who suffer harm from trial participation | n/a |
| Dissemination policy: trial results | #31a | Plans for investigators and sponsor to communicate trial results to participants, healthcare professionals, the public, and other relevant groups (eg, via publication, reporting in results databases, or other data sharing arrangements), including any publication restrictions | 2 |
| Dissemination policy: authorship | #31b | Authorship eligibility guidelines and any intended use of professional writers | n/a |
| Dissemination policy: reproducible research | #31c | Plans, if any, for granting public access to the full protocol, participant-level dataset, and statistical code | n/a |
| **Appendices** | | | |
| Informed consent materials | #32 | Model consent form and other related documentation given to participants and authorised surrogates | 18 |
| Biological specimens | #33 | Plans for collection, laboratory evaluation, and storage of biological specimens for genetic or molecular analysis in the current trial and for future use in ancillary studies, if applicable | n/a |

None The SPIRIT checklist is distributed under the terms of the Creative Commons Attribution License CC-BY-ND 3.0. This checklist can be completed online using https://www.goodreports.org/, a tool made by the EQUATOR Network in collaboration with Penelope.ai

For peer review only

BMJ Open

# Optimizing implementation strategies of the first scale-up of a primary care psychological intervention for common mental disorders in Sub-Saharan Africa: A Mixed Methods Study protocol for the Optimized Friendship Bench (OptFB)



| | |
|---|---|
| Journal: | *BMJ Open* |
| Manuscript ID | bmjopen-2020-045481.R1 |
| Article Type: | Protocol |
| Date Submitted by the Author: | 22-Mar-2021 |



| | |
|---|---|
| Complete List of Authors: | Verhey, Ruth ; University of Zimbabwe, Research Support Centre; Friendship Bench<br>Chitiyo, Charmaine; Friendship Bench Zimbabwe<br>Mboweni, Sandra; Friendship Bench Zimbabwe<br>Chiriseri, Ephraim; Friendship Bench Zimbabwe<br>Chibanda, Dixon; Friendship Bench Zimbabwe; University of Zimbabwe, Research Support Centre<br>Healey, Andy; King's College London, IOPPN<br>Wagenaar, Bradley; University of Washington, Department of epidemiology; University of Washington, Department of global health<br>Araya, Ricardo; Centre for Global Mental Health and Primary Care Research, |



| | |
|---|---|
| <b>Primary Subject Heading</b>: | Mental health |
| Secondary Subject Heading: | Global health, Communication |
| Keywords: | MENTAL HEALTH, PRIMARY CARE, PUBLIC HEALTH, QUALITATIVE RESEARCH |
| | |


SCHOLARONE™
Manuscripts

*I, the Submitting Author has the right to grant and does grant on behalf of all authors of the Work (as defined in the below author licence), an exclusive licence and/or a non-exclusive licence for contributions from authors who are: i) UK Crown employees; ii) where BMJ has agreed a CC-BY licence shall apply, and/or iii) in accordance with the terms applicable for US Federal Government officers or employees acting as part of their official duties; on a worldwide, perpetual, irrevocable, royalty-free basis to BMJ Publishing Group Ltd ("BMJ") its licensees and where the relevant Journal is co-owned by BMJ to the co-owners of the Journal, to publish the Work in this journal and any other BMJ products and to exploit all rights, as set out in our licence.*

*The Submitting Author accepts and understands that any supply made under these terms is made by BMJ to the Submitting Author unless you are acting as an employee on behalf of your employer or a postgraduate student of an affiliated institution which is paying any applicable article publishing charge ("APC") for Open Access articles. Where the Submitting Author wishes to make the Work available on an Open Access basis (and intends to pay the relevant APC), the terms of reuse of such Open Access shall be governed by a Creative Commons licence – details of these licences and which Creative Commons licence will apply to this Work are set out in our licence referred to above.*

*Other than as permitted in any relevant BMJ Author's Self Archiving Policies, I confirm this Work has not been accepted for publication elsewhere, is not being considered for publication elsewhere and does not duplicate material already published. I confirm all authors consent to publication of this Work and authorise the granting of this licence.*

For peer review only

Optimizing implementation strategies of the first scale-up of a primary care psychological intervention for common mental disorders in Sub-Saharan Africa: A Mixed Methods Study protocol for the Optimized Friendship Bench (OptFB)

Author list:
Ruth Verhey [a,b]
Charmaine Chitiyo [a]
Sandra Mboweni [a]
Ephraim Chiriseri [a]
Dixon Chibanda [a,b,d]
Andy Healey [c]
Bradley H. Wagenaar [e,f]
Ricardo Araya [c]

[a] Friendship Bench Zimbabwe
[b] University of Zimbabwe
[c] King's College, London, UK
[d] London school of hygiene and tropical medicine, LSHTM, UK
[e] Department of Global Health, University of Washington, Settle, WA, USA
[f] Department of Epidemiology, University of Washington, Seattle, WA, USA

Abstract word count: 281
Body word count: 3650

Corresponding author:
Ruth Verhey
Address: 4 Weale Road, Milton Park Harare Zimbabwe
Tel: +263773857376 Email: ruth.verhey@friendshipbench.io


For peer review only - http://bmjopen.bmj.com/site/about/guidelines.xhtml

## Abstract

Introduction: Common mental disorders (CMDs) are a leading cause of disability globally. CMDs are highly prevalent in Zimbabwe and have been addressed by an evidence-based, task-shifting psychological intervention called the Friendship Bench (FB). The task-shifted FB program guides clients through problem solving therapy. It was scaled-up across 36 implementation sites in Zimbabwe in 2016.

Methods and analysis: This study will employ a mixed-methods framework. It aims to: (1) Use quantitative survey methodologies organized around the RE-AIM evaluation framework to assess the current scale-up of the FB intervention and classify 36 clinics according to levels of performance; (2) Use qualitative focus group discussions and semi-structured interviews organized around the Consolidated Framework for Implementation Research (CFIR) to analyze determinants of implementation success, as well as elucidate heterogeneity in implementation strategies through comparing high- and low-performing clinics; and (3) Use the results from aims 1 and 2 to develop strategies to optimize the Friendship Bench intervention and apply this model in a cluster randomized controlled trial to evaluate potential improvements among low-performing clinics. The trial will be registered with the Pan African Clinical Trial Registry (www.pactr.org). The planned randomized controlled trial for the third research aim will be registered after completing aims one and two because the intervention is dependent on knowledge generated during these phases.

Ethics and dissemination: The research protocol received full authorization from the Medical Research Council of Zimbabwe (MRCZ A/242). It is anticipated that changes in data collection tools and consent forms will take place at all three phases of the study and approval from MRCZ will be sought. All interview partners will be asked for informed consent. The research team will prioritize open access publications to disseminate research results.




For peer review only - http://bmjopen.bmj.com/site/about/guidelines.xhtml

## Strengths and limitations of this study

- Few evidence-based psychological interventions offered at primary health care level have been successfully scaled-up in Sub-Saharan Africa; this study is designed to deliver detailed knowledge about factors that influence the scale-up of a primary care psychological intervention (the Friendship Bench) in an African setting.
- Two widely used implementation science models, RE-AIM and CFIR, will be used to evaluate the implementation of this intervention, which was scaled up in 2016.
- This study focuses on evaluating the scaling up of evidence-based interventions and developing and testing implementation strategies to potentially optimize the routine delivery of the Friendship Bench.
- A limitation is that comprehensive implementation data is only collected three years after the scale up exercise.

Key words: Friendship Bench, Optimization, common mental disorders, CFIR, RE-AIM, Low- and middle-income countries

For peer review only

## 1. Introduction:

In the past 10 years, it has become apparent that mental, neurological and substance use disorders (MNS) are among the leading causes of the global disease burden [1-3]. Research has shown that 4 out of every 10 people in low-and-middle-income countries (LMICs) suffer from mental disorders (de Boer et al. 2008, World Health Organization, 2009a) and evidence-based mental health interventions have become a focus of research and interest [4]. It has been observed that the poor are disproportionately affected by mental disorders [5 6]. Less than 5% of people living in some LMIC receive any adequate treatment for mental health disorders [7 8 9]. Particularly in low- and



For peer review only - http://bmjopen.bmj.com/site/about/guidelines.xhtml

middle-income countries (LMIC) the lack of resources, especially trained mental health professionals, causes sub-optimal detection and management of CMD [10-12]. Worldwide, efforts have been made to create sustainable and affordable mental health interventions in primary care [13-18]. In a recent systematic review, only four studies were detected that had evaluated the implementation of a depression intervention scaled-up in routine care [19]. As it stands, the benefit of these evidence-based interventions is not yet reaching those populations most at need across LMICs.

Zimbabwe, a country in Southern Africa with a population of 13 million has a large treatment gap for MNS. Studies show that over 30% of primary health care (PHC) users need mental health care services for mostly common mental disorders (CMD) and only 5% of these receive appropriate care [20]. Untreated CMD can also lead to worsening of clinical outcomes in chronic conditions such as HIV [21] and negatively affect economic outcomes too [5]. The Friendship Bench (FB) was developed in response to the existing treatment gap for mental health care in Zimbabwe and tested for its efficacy in a cluster randomized controlled trial (RCT) [22].

This task-shifted intervention is delivered by trained and supervised lay health workers (LHWs) who deliver problem solving therapy (PST) [23] on a bench located in primary health care clinics. In 2016, the FB intervention was scaled-up across Harare, Gweru and Chitungwiza and surrounding peri-urban communities in collaboration with the respective City Health departments [24]. The FB program was established in 72 City Health PHC clinics that are established in 36 sites (different clinic types can be found in the same site). This scaling-up exercise involved the training of more than 300 LHWs in the 3 cities in Zimbabwe [24]. Maintenance funding for FB activities is provided by the City Health department.

All lay health workers (LHWs) working for the FB PHC clinics in Harare, Gweru and Chitungwiza received the standard manualized training and supervision. While existing scientific evidence has shown that under ideal randomized trial conditions the FB intervention leads to clinically-significant reductions in symptoms, little implementation research has been carried out regarding the performance of Friendship Bench under routine conditions as the model is being further scaled-up across Zimbabwe.



For peer review only - http://bmjopen.bmj.com/site/about/guidelines.xhtml

This study will be of interest to implementation scientists, policymakers, and researchers working to scale-up primary care psychological interventions in low- and middle-income countries (LMICs) globally. Results from this study have the potential to inform future scale-up and maintenance of task-shared psychological interventions into routine Ministry of Health primary care settings.

## 2. Overall Study Goal

This research uses a mixed-methods study design and widely-used implementation frameworks to systematically analyze the performance of clinics, determinants of this performance, including implementation strategies that might differentiate high- versus low-performing clinics, and develop and test an enhanced implementation strategy to improve the performance of clinics in three cities in Zimbabwe. The study is designed to be conducted in three phases with corresponding aims.

Firstly (aim 1), we plan to examine how the FB is performing under real-world implementation conditions and classify existing clinics with FB into high- versus low-performing sites using differences in RE-AIM outcomes [25 26].

Secondly (aim 2), we will analyse the determinants of heterogeneity in the results of phase 1 comparing high- versus low-performing clinics, mainly using the CFIR framework [27] and rigorously documenting changes to the original FB protocol and current implementation strategies in use.

Thirdly (aim 3), we will develop and test an optimized package of FB implementation strategies based on the results of phase 2 and measure the improvement among low performing clinics using RE-AIM outcomes.

## 3. Study setting:

The study will be conducted in primary health care clinics (PHC) in Harare, Gweru, and Chitungwiza.

Most of the clinics in the 3 cities are located in comparable areas which are characterized by high population density and informal income generating activities often occurring in the vicinity of the clinics. Depending on their size, PHC clinics serve between 20,000-80,000 people from the most socio-economically disadvantaged sectors of the population. Clinics are differentiated into poly, satellite, and family health service clinics according to the size of the clinic and the range of services offered.



For peer review only - http://bmjopen.bmj.com/site/about/guidelines.xhtml

The most comprehensive services are offered in a Polyclinic such as pre-, post- and perinatal care, opportunistic infections (for example TB treatment), and specialized NGO-based programs (HIV testing and management, male circumcision, communicable disease awareness). Satellite and Family health clinics (FHS) offer less services. Medical doctors are not permanently present but hold clinics on specific days in poly clinics. This influences the clinic user population's composition on these particular days (for example HIV clinic day).

Clinics in Harare, Chitungwiza and Gweru are grouped and located in the same geographical facility and these are counted as one Friendship Bench implementation site. Data will be collected in 36 implementation sites (n=28 in Harare; n=4 in Gweru; n=4 in Chitungwiza). Of these 26 Poly clinics, six are FHS and four satellite clinics (see figure 1).

Figure 1: Clinic type distribution for Harare, Gweru and Chitungwiza

Depending on their size and catchment area, FB implementation sites have between one (1) and fourteen (14) LHWs who deliver the FB intervention on benches in the clinic premises during clinic opening times. Clinic users are informed about the about FB services and mental health through group or individual talks in the clinic's waiting areas. Community members are also directly in contact with LHWs during outreach activities in the community.

## 4. Methods

This study proposes a rigorous analysis of the multiple interconnecting factors using two internationally recognized implementation research methods – the RE-AIM model [26] and the CFIR [27] which will be described in more detail below. Both conceptual frameworks have been used widely in implementation research for health care delivery in order to deepen the understanding and evaluation of interventions such as the Friendship Bench. The study has three research aims which are linked contextually to each other and are described in detail below.

Patient and Public Involvement



For peer review only - http://bmjopen.bmj.com/site/about/guidelines.xhtml

Patients and/or the public will be involved in the stakeholder meetings, they were not involved in the design, nor will they be involved in the study conduct, or reporting, or dissemination plans of this research project.

---

## 4.1 Methods Aim 1

A thorough analysis of the existing routine health information system data collected by the Harare, Gweru and Chitungwiza City Health authorities will be carried out to learn about the Friendship Bench activities at individual clinic level. This data consists of user numbers, age, gender, HIV status, clients' screening tool scores pre- and post-intervention as well as complete use of screening tool, and number of sessions.

We will use the RE-AIM evaluation framework to evaluate the current implementation performance of the FB intervention after three years of implementation experience. Routinely collected data will be used to assess the FB intervention's real-world and pragmatic performance: Reach, Effectiveness, Adoption, Implementation, and Maintenance. The research team which consists of experienced global mental health researchers and clinicians will develop indicators for each of the RE-AIM domains using the www.re-aim.org website to support us and base our decisions on expert consensus and availability of data. These indicators will then be used to design a questionnaire to guide the RE-AIM related data collection. Each indicator will comprise a numerator and a denominator populated with data collected from the clinic records and the planned observations.

The data on the FB implementation will be analyzed for each of the 36 participating clinics. Routinely collected data includes clinical registries for both nurses and LHWs and data from the FB Register (commonly known as the "green book") where the LHWs record beneficiary information.

In addition, LHWs will be observed during all aspects of their work, including giving health talks, interacting with clients, and delivering the FB intervention. We will observe and record whether all FB related tools such as questionnaires and intervention tools are used.



For peer review only - http://bmjopen.bmj.com/site/about/guidelines.xhtml

In order to collect additional necessary data for AIM 1, key respondents will be interviewed using a questionnaire that will be developed by the research team.
We plan to interview at least 2 LHWs per clinic and in clinics with more than 2 LHWs, we will interview 50% of the present LHWs by randomly selecting them. Papers with their names will be put in a container from which a RA will pull out the appropriate number in the LHWs' presence. We will always interview the supervisor LHW of each clinic if this position is taken in a particular clinic. We will also interview the nurse in charge in every clinic and the associated district health promoting officers (DHPOs) (n=10). Data will be collected from June to September 2019 in all participating sites.

The data collection will be carried out by two research coordinators who will lead two teams of four trained and supervised research assistants (RAs). The teams will visit each clinic for two days. The clinics will be sensitized about the FB team visit a week prior. The research assistants will be trained to interview, to observe and record the FB related activities in the clinic and how to enter the data digitally using tablet computers. They will be trained on data checking, cleaning and uploading.

Furthermore, we are planning to audio-record FB sessions with consenting clients (two per site, n=72). We will approach, where possible, all incoming clients seeking services and ask them for informed consent to allow us to record their session with the FB LHWs. We aim to record as many as possible but at least 2 per site.
The recordings will be translated, transcribed and rated according to the Friendship Bench fidelity checklist.
The FB fidelity checklist assesses for communication skills of the counselor, the level of psychoeducation that is done, and the adherence to the problem-solving therapy steps that the FB counselor is trained to deliver (see Supplementary Appendix A for full fidelity checklist which was developed for the RCT [22]). The assessments of audio recordings will be done by trained FB research team members who will prepare an audio-recorder which will be left with the FB counselor after a client has given consent. The audio recording device will be retrieved by the research assistant when the LHW has indicated that the session is done.
In the event that no clients come to the clinic on both days that the FB team visits the site or no client consents to have their session audio-recorded, this will be entered as


For peer review only - http://bmjopen.bmj.com/site/about/guidelines.xhtml

missing. Due to logistic and financial constraints a repeat visit to a particular clinic will not be possible.

All respondents will be asked to answer the questions with regards to FB activities in the past month. According to their position with regards to FB activities, questions might be formulated slightly differently.

The questionnaires will be administered using tablet computers (Lenovo), all observational data will be entered digitally after their correctness has been ascertained by asking interviewees to show evidence as applicable. Questionnaires and observation guides are programmed into the tablets using Kobotoolbox (https://www.kobotoolbox.org) which is a data collection tool. Collected data will be cleaned and uploaded daily to a password secured server.

The research team will also observe FB specific activities such as health and 'mobilization' talks that are given by the clinic staff including the LHWs whilst patients are waiting to be seen.

A stakeholder meeting will be held once Aim 1 data is completed and the data is analyzed. At this meeting, the research team will present the results from Aim 1 and discuss potential reasons why we might see the differences in implementation across sites with stakeholders. This meeting will be attended by all relevant clinic staff, health authority officers as well as clients. Information from stakeholders will be used to select and prioritize CFIR constructs to include in qualitative interview guides for Aim 2.

**4.1.1 Data Analysis Aim 1**

The goal of Aim 1 is to classify the 36 FB implementation sites on their performance based on the RE-AIM outcomes. Our methods will follow similar classification efforts previously published [28]. Clinics will be first ranked according to their performance within each individual measure. Clinics score on all indicators within one construct (for example reach) will be averaged. For each of the RE-AIM constructs, every clinic will thus have an averaged ranking.

For peer review only

These domain-based rankings will be averaged per clinic rankings giving an overall ranking by calculating simple means of all domain rankings. This procedure will be carried out by two independent individuals and any differences will lead to a redoing of the process. In case of same outcomes for clinics, we will treat these particular clinics as being on the same rank. This will give us a final composite rank for each clinic which will be used to determine the 10 highest and 10 lowest performing clinics that will be qualitatively assessed in Aim 2.

**4.2 Methods Aim 2**

With the aim to understand the determinants of implementation success, as well as differences in implementation strategies employed, Aim 2 will utilize focus-group discussions organized around the Consolidated Framework for Implementation Research (CFIR) [29-31]. Through these qualitative methods, we aim to gain a deeper understanding of the factors that contribute to the successful implementation comparing high- with low-performing clinics. The CFIR framework focuses on an overview of potential multi-level determinants of health care delivery. It was designed to help understand integrated implementation determinants across multiple levels (clients; implementers; organizations; contexts; processes).

For the present study, we will focus on determinants of implementation success, taking lessons from both high- and low-performing clinics to inform the development of an improved package of implementation strategies targeting identified barriers.
Focus group discussions (FDGs) with key informants (LHWs, nurses, DHPOs, clients) of the 10 high and 10 low performing clinics will be carried out by trained qualitative researchers. The FB specific interview guides for these group discussions and interviews will be developed by the study team in a sequence of internal project meetings using the online technical support website www.cfirguide.org. The results of Aim 1 will guide us in designing the interview guides for the focus group discussions.
The outcome of the stakeholders meeting in which we present the results of Aim 1 will also give us insight on the importance of constructs which we will take into account when designing the CFIR interview guides.
Interview guides will be translated into the local language Shona and all group discussions will be audio-recorded, transcribed and translated to English. All discussions will be held in the local language.


For peer review only - http://bmjopen.bmj.com/site/about/guidelines.xhtml

The FGD participants will be selected from all 10 low and high performing clinics, respectively. We will interview LHWs, nurses, DHPOs in their role as implementers as well as clients as recipients of the intervention. Nurses and DHPOs will be invited to joined meetings. We will conduct FGDs for all available LHWs at every selected clinic. We will ask the selected LHWs to purposively suggest 2 clients each, whom we will then invite to FGDs in each of the selected clinics. In case a client declines participation, we will ask for another suggestion.

Focus group discussions will take place in clinics or, if not possible, in the Friendship Bench office in Harare.

For peer review only

**4.2 1 Data Analysis Aim 2**

CFIR analyses will follow the original Damschroder methodology previously published [30]. Briefly, two independent local Zimbabwean reviewers will code each FGD transcript according to the selected CFIR constructs. Differences will be discussed and revised until final codes are agreed on. Facility-level case memos will be organized by the relevant CFIR construct, using each new transcript to confirm and refine statements until all transcripts are coded. This process will be closely supported by the whole research team. Each clinic will have two case memos, one for LHWs and other implementers and one for clients.

Using case memos and supporting transcripts, the same two coders will independently rate CFIR constructs on valence (X (mixed); 0 (neutral); + (construct has a positive effect on implementation) or – (construct has a negative influence on implementation). Once drafted, the entire research team will meet and use a deliberated consensus to finalize memos, constructs, and valence. These data will be mapped on a matrix template with the goal of identifying constructs that differ between facilities with high and low performance to identify factors relevant for the success of the implementation. Analyses will progress with visual inspection of patterns in constructs and valence by high versus low performing clinics, as well as examining median and mean valence by high versus low performing clinics. Once distinguishing constructs are identified, the team will re-review case memos and coded transcripts to gather more information on constructs.

**4.3 Aim 3**



For peer review only - http://bmjopen.bmj.com/site/about/guidelines.xhtml

In Aim 3, we will develop a package of optimized Friendship Bench (OptFB) implementation strategies matched to key barriers identified in the previous phases of this study. Using CFIR data on barriers / facilitators to high-quality FB implementation, we will use the CFIR-ERIC matching tool to examine and select implementation strategies to address key CFIR constructs discriminating between high and low performing clinics in Aim 1 (https://cfirguide.org/choosing-strategies/) [32 33]. Once a preliminary list is developed by our team, the CFIR-Expert Recommendation for Implementation Change (ERIC) matching tool [32] will be used to prioritize those strategies that are found to be most likely to address CFIR barriers in low-performing clinics [33 34].

We will engage in a participatory stakeholder Delphi rating exercise to select specific strategies. This will be followed by the research team specifying and tailoring the strategies for the Zimbabwean context by including the additional information gained from the stakeholders. Aspects of feasibility, affordability and effectiveness will guide this process in order for the package to be meaningful and effective [35]. Strategies currently in use by high performing clinics will be also considered for the optimized Friendship Bench (OptFB) implementation strategies.

This OptFB package or intervention of improved strategies will be tested in low-performing clinics. Ongoing RE-AIM data is being collected on a monthly basis in each clinic. Using these data on RE-AIM outcomes, we will re-classify clinics using a similar process as in Aim 1. We will then identify the 18 lowest performing clinics and randomly select 12 clinics to deliver the OptFB and 6 to act as control clinics over a period of 6 months. The primary outcome will be a composite measure of RE-AIM indicators (Reach, Effectiveness, Adoption and Implementation and Maintenance) estimated at 6-month after the commencement of the implementation of the OptFB intervention. We will estimate changes in this composite measure of implementation before and at 6 months after starting the delivery of OptFB in all clinics. We will compare the difference in means or proportions between the clinics receiving the OptFB and the control clinics using the routinely collected data. Secondary outcomes will examine performance of each of the RE-AIM outcomes separately and clinical effectiveness results at individual level. The latter will be based on individual scores ~~to~~ on the SSQ on a minimum of 20 random individuals per clinic during the 6-month period.

For peer review only

No sample size calculation has been estimated since there are no previous studies on which to estimate an effect size, the number of clinics is small, and the main outcomes are averaged data representing clusters. Nonetheless, we expect to see larger improvements in the RE-AIM composite index score in the clinics receiving OptFB compared to the control clinics over the 6 months. As a secondary outcome measure, clinical effectiveness will be assessed based on changes on SSQ scores from baseline to 6 months for a sample of 360 individuals (18 clinics with 20 individuals each), but we do not expect this sample would have enough power to detect small differences in effectiveness across the two group of clinics. Thus, comparisons on clinical effectiveness must be considered purely descriptive and exploratory and interpreted with caution. In any case, the main outcomes of interest in this study are implementation outcomes subsumed under the domains included in the RE-AIM framework.

**4.3.1 Data analysis Aim 3**

We will use a difference-in-differences analysis comparing the groups over time. Means or proportions on outcome data will be compared across groups using descriptive statistics. Regression models will be used to estimate the effect of the intervention on the main outcomes. General estimating equations with robust standard errors will be used to control for clustering. Potential confounders will be determined a priori and included in the regression models. Standard errors, confidence intervals, and p-values will be obtained. A similar secondary analysis will be conducted with the secondary outcome measures.

**4.4 Health economic analysis**

Site-level data will be collected on fidelity to the OptFB implementation strategies, along with activities and resource inputs required to deliver improvement strategies and OptFB delivery costs. Economic modelling will be used to combine this information with data and evidence on clinical impact and implementation effectiveness to evaluate the cost-effectiveness of the OptFB program [36].

We will also revisit clinics and re-engage with stakeholders in ~~into~~ FGD to explore level of change in the identified CFIR domains in the intervention arm clinics.

For peer review only



For peer review only - http://bmjopen.bmj.com/site/about/guidelines.xhtml

After completion of the trial, the strategy will also be implemented in the control arm clinics to increase the overall performance in all of participating lower performing clinics.

## 5. Discussion

This study will contribute to the knowledge about scaling up of an evidence-based task-shifted intervention in a LMIC. This is a unique opportunity to analyze the Friendship Bench in a real-world setting. As mentioned above, not many interventions have been scaled up from LMICs and therefore there is a dearth of information on how implementation strategies can be used in order to ensure a strong scaling up. With this study we hope to learn which barriers and enablers are at play in the FB scale up process. This is particularly important for us as we are expanding the FB services throughout Zimbabwe and beyond to meet the population's needs for accessible and acceptable mental health care. This effort has to be undertaken with the aim of having high fidelity to the program while considering contextual aspects. Using implementation science principles will help us to give theoretical justification and describe specifications for application for those implementation strategies that we will devise after having gone through the different stages of this research process. Evidence-based, clear and applicable guidelines of how to implement our evidence-based intervention in primary health care settings will be created and can then subsequently be used to ensure a strong implementation of FB.

## 6. Ethics and dissemination

This research protocol has been approved by the Medical Research Council Zimbabwe (MRCZ), MRCZ/A/2428 and the Joint Research Council (JREC), 79/19. Results will be disseminated in peer-reviewed journals and conferences.

For peer review only

## References

1. Vigo D, Thornicroft G, Atun R. Estimating the true global burden of mental illness. *Lancet Psychiatry* 2016;3(2):171-8. doi: 10.1016/S2215-0366(15)00505-2 [published Online First: 2016/02/07]
2. Kessler RC, Aguilar-Gaxiola S, Alonso J, et al. The global burden of mental disorders: an update from the WHO World Mental Health (WMH) surveys. *Epidemiol Psichiatr Soc* 2009;18(1):23-33. doi: 10.1017/s1121189x00001421 [published Online First: 2009/04/22]
3. Whiteford HA, Ferrari AJ, Degenhardt L, et al. The global burden of mental, neurological and substance use disorders: an analysis from the Global Burden of Disease Study 2010. *PLoS One* 2015;10(2):e0116820. doi: 10.1371/journal.pone.0116820 [published Online First: 2015/02/07]
4. Steel Z, Marnane C, Iranpour C, et al. The global prevalence of common mental disorders: a systematic review and meta-analysis 1980–2013. *International journal of epidemiology* 2014:dyu038.
5. Lund C, De Silva M, Plagerson S, et al. Poverty and mental disorders: breaking the cycle in low-income and middle-income countries. *The Lancet* 2011;378(9801):1502-14.
6. Evans-Lacko S, Aguilar-Gaxiola S, Al-Hamzawi A, et al. Socio-economic variations in the mental health treatment gap for people with anxiety, mood, and substance use disorders: results from the WHO World Mental Health (WMH) surveys. *Psychological medicine* 2018;48(9):1560-71. doi: 10.1017/S0033291717003336 [published Online First: 2017/11/28]
7. Wang PS, Aguilar-Gaxiola S, Alonso J, et al. Use of mental health services for anxiety, mood, and substance disorders in 17 countries in the WHO world mental health surveys. *The Lancet* 2007;370(9590):841-50.
8. Mojtabai R, Olfson M, Sampson NA, et al. Barriers to mental health treatment: results from the National Comorbidity Survey Replication. *Psychological medicine* 2011;41(8):1751-61. doi: 10.1017/S0033291710002291 [published Online First: 2010/12/08]
9. Kohn R, Saxena S, Levav I, et al. The treatment gap in mental health care. *Bull World Health Organ* 2004;82(11):858-66. doi: /S0042-96862004001100011 [published Online First: 2005/01/11]
10. Prince M, Patel V, Saxena S, et al. No health without mental health. *The lancet* 2007;370(9590):859-77.
11. Alonso J, Liu Z, Evans-Lacko S, et al. Treatment gap for anxiety disorders is global: Results of the World Mental Health Surveys in 21 countries. *Depress Anxiety* 2018;35(3):195-208. doi: 10.1002/da.22711 [published Online First: 2018/01/23]
12. Wang PS, Angermeyer M, Borges G, et al. Delay and failure in treatment seeking after first onset of mental disorders in the World Health Organization's World Mental Health Survey Initiative. *World Psychiatry* 2007;6(3):177-85. [published Online First: 2008/01/12]
13. Araya R, Rojas G, Fritsch R, et al. Treating depression in primary care in low-income women in Santiago, Chile: a randomised controlled trial. *Lancet* 2003;361(9362):995-1000. doi: 10.1016/S0140-6736(03)12825-5
14. Patel V, Thornicroft G. Packages of care for mental, neurological, and substance use disorders in low-and middle-income countries: PLoS Medicine Series. *PLoS medicine* 2009;6(10):e1000160.



For peer review only - http://bmjopen.bmj.com/site/about/guidelines.xhtml

15. Chatterjee S, Chowdhary N, Pednekar S, et al. Integrating evidence-based treatments for common mental disorders in routine primary care: feasibility and acceptability of the MANAS intervention in Goa, India. *World Psychiatry* 2008;7(1):39-46.
16. Rahman A, Malik A, Sikander S, et al. Cognitive behaviour therapy-based intervention by community health workers for mothers with depression and their infants in rural Pakistan: a cluster-randomised controlled trial. *The Lancet* 2008;372(9642):902-09.
17. Bolton P, Bass J, Neugebauer R, et al. Group interpersonal psychotherapy for depression in rural Uganda: a randomized controlled trial. *Jama* 2003;289(23):3117-24.
18. Lund C, Alem A, Schneider M, et al. Generating evidence to narrow the treatment gap for mental disorders in sub-Saharan Africa: rationale, overview and methods of AFFIRM. *Epidemiol Psychiatr Sci* 2015;24(3):233-40. doi: 10.1017/S2045796015000281 [published Online First: 2015/04/03]
19. Wagenaar BH, Hammett, W.H., Jackson, C., Atkins, D.L., Belus, J.M. and Kemp, C.G. Implementation outcomes and strategies for depression interventions in low-and middle-income countries: a systematic review. . *Global Mental Health* 2020;7
20. Chibanda D, Mesu P, Kajawu L, et al. Problem-solving therapy for depression and common mental disorders in Zimbabwe: piloting a task-shifting primary mental health care intervention in a population with a high prevalence of people living with HIV. *BMC public health* 2011;11:828. doi: 10.1186/1471-2458-11-828 [published Online First: 2011/10/28]
21. Antelman G, Kaaya S, Wei R, et al. Depressive symptoms increase risk of HIV disease progression and mortality among women in Tanzania. *Journal of acquired immune deficiency syndromes* 2007;44(4):470-7. doi: 10.1097/QAI.0b013e31802f1318
22. Chibanda D, Weiss HA, Verhey R, et al. Effect of a Primary Care-Based Psychological Intervention on Symptoms of Common Mental Disorders in Zimbabwe: A Randomized Clinical Trial. *JAMA* 2016;316(24):2618-26. doi: 10.1001/jama.2016.19102 [published Online First: 2016/12/28]
23. Chibanda D, Bowers T, Verhey R, et al. The Friendship Bench programme: a cluster randomised controlled trial of a brief psychological intervention for common mental disorders delivered by lay health workers in Zimbabwe. *Int J Ment Health Syst* 2015;9:21. doi: 10.1186/s13033-015-0013-y [published Online First: 2015/01/01]
24. Chibanda D, Verhey R, Munetsi E, et al. Scaling up interventions for depression in sub-Saharan Africa: lessons from Zimbabwe. *Glob Ment Health (Camb)* 2016;3:e13. doi: 10.1017/gmh.2016.8 [published Online First: 2016/04/11]
25. Glasgow RE, Vogt TM, Boles SM. Evaluating the public health impact of health promotion interventions: the RE-AIM framework. *Am J Public Health* 1999;89(9):1322-27.
26. Glasgow RE, Vogt TM, Boles SM. Evaluating the public health impact of health promotion interventions: the RE-AIM framework. *American journal of public health* 1999;89(9):1322-7. doi: 10.2105/ajph.89.9.1322 [published Online First: 1999/09/04]
27. Damschroder LJ, Aron DC, Keith RE, et al. Fostering implementation of health services research findings into practice: a consolidated framework for advancing implementation science. *Implement Sci* 2009;4:50. doi: 10.1186/1748-5908-4-50 [published Online First: 2009/08/12]
28. Farris RP, Will JC, Khavjou O, et al. Beyond effectiveness: evaluating the public health impact of the WISEWOMAN program. *American journal of public health* 2007;97(4):641-47.



For peer review only - http://bmjopen.bmj.com/site/about/guidelines.xhtml

29. Breimaier HE, Heckemann B, Halfens RJ, et al. The Consolidated Framework for Implementation Research (CFIR): a useful theoretical framework for guiding and evaluating a guideline implementation process in a hospital-based nursing practice. *BMC Nurs* 2015;14:43. doi: 10.1186/s12912-015-0088-4 [published Online First: 2015/08/14]
30. Damschroder LJ, Lowery JC. Evaluation of a large-scale weight management program using the consolidated framework for implementation research (CFIR). *Implement Sci* 2013;8:51. doi: 10.1186/1748-5908-8-51 [published Online First: 2013/05/15]
31. Damschroder LJ, Aron DC, Keith RE, et al. Fostering implementation of health services research findings into practice: a consolidated framework for advancing implementation science. *Implementation science* 2009;4(1):50.
32. Powell BJ, Waltz TJ, Chinman MJ, et al. A refined compilation of implementation strategies: results from the Expert Recommendations for Implementing Change (ERIC) project. *Implement Sci* 2015;10:21. doi: 10.1186/s13012-015-0209-1 [published Online First: 2015/04/19]
33. Waltz TJ, Powell BJ, Fernandez ME, et al. Choosing implementation strategies to address contextual barriers: diversity in recommendations and future directions. *Implement Sci* 2019;14(1):42. doi: 10.1186/s13012-019-0892-4 [published Online First: 2019/05/01]
34. Godbee K, Gunn J, Lautenschlager NT, et al. Refined conceptual model for implementing dementia risk reduction: incorporating perspectives from Australian general practice. *Aust J Prim Health* 2020;26(3):247-55. doi: 10.1071/PY19249 [published Online First: 2020/05/28]
35. Cooper LA, Hill MN, Powe NR. Designing and evaluating interventions to eliminate racial and ethnic disparities in health care. *J Gen Intern Med* 2002;17(6):477-86. doi: 10.1046/j.1525-1497.2002.10633.x [published Online First: 2002/07/23]
36. Briggs A, Sculpher, M., & Claxton, K.  . Decision modelling for health economic evaluation. Oxford: Oup Oxford 2006.

For peer review only

## Footnotes

### Contributors

RA designed the study and received the grant. RA is the overall PI. RV and DC are leading the study locally. CC will have oversight as project coordinator together with SM as her assistant over the data collection in all phases. BW is leading on the implantation science aspects. EC is conducting data cleaning and assisting with analysis. AH is leading on the health economics analysis. All authors will contribute to the development of questionnaires, interview guides and the strategies for the intervention. RV wrote the first version of the protocol paper. All authors contributed by critically reviewing all further drafts and approving of the final paper.

### Funding

This work was supported by Global Alliance for Chronic Diseases (GACD) through the Medical Research Council Grant number: MRC UKRI MR/S004270/1.

**Competing interests** None declared.

**Patient consent for publication** Not required.



For peer review only - http://bmjopen.bmj.com/site/about/guidelines.xhtml

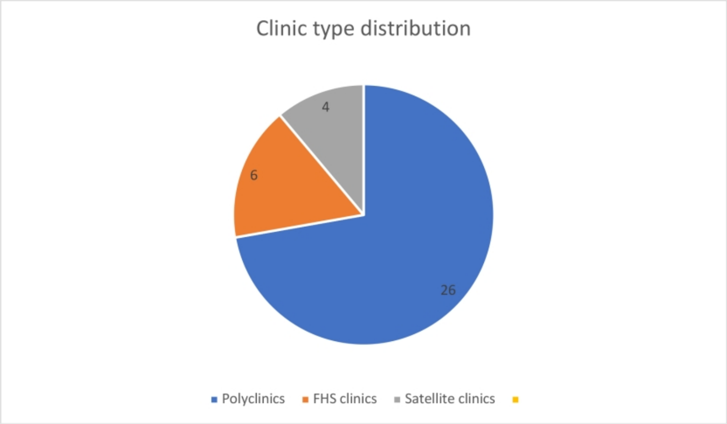


Figure 1: Clinic type distribution for Harare, Gweru and Chitungwiza

**Table 1. Checklist for audio-recorded sessions**

## LHW FIDELITY CHECKLIST

**LHW Name_________________________ Client's Name___________________________**
**Date / / Site Code** ☐☐

| *(Tick where appropriate)* | | YES | NO |
|---|---|---|---|
| 1. | LHW introduced self to client and asked client to introduce self | ☐ | ☐ |
| 2. | Psycho-education done properly | | |
| | a. Linked HIV to kufungisisa | ☐ | ☐ |
| | b. Adherence | ☐ | ☐ |
| | c. Diet advise done | ☐ | ☐ |
| 3. | Problems presented by client | ☐ | ☐ |
| 4. | LHW listening and acknowledging | ☐ | ☐ |
| 5. | LHW gives summary of problems | ☐ | ☐ |
| 6. | Client selected problem, not LHW | ☐ | ☐ |
| 7. | LHW and client discuss problem identified by client | ☐ | ☐ |
| 8. | Client identifies solutions to the problem identified | ☐ | ☐ |
| 9. | LHW and client identify task for client to work with | ☐ | ☐ |
| 10. | Session closure and next correct review date | ☐ | ☐ |
| | ***Total*** | ____/10 | ___/10 |

For peer review only

# Reporting checklist for protocol of a clinical trial.

Based on the SPIRIT guidelines.

## Instructions to authors

Complete this checklist by entering the page numbers from your manuscript where readers will find each of the items listed below.

Your article may not currently address all the items on the checklist. Please modify your text to include the missing information. If you are certain that an item does not apply, please write "n/a" and provide a short explanation.

Upload your completed checklist as an extra file when you submit to a journal.

In your methods section, say that you used the SPIRITreporting guidelines, and cite them as:

Chan A-W, Tetzlaff JM, Gøtzsche PC, Altman DG, Mann H, Berlin J, Dickersin K, Hróbjartsson A, Schulz KF, Parulekar WR, Krleža-Jerić K, Laupacis A, Moher D. SPIRIT 2013 Explanation and Elaboration: Guidance for protocols of clinical trials. BMJ. 2013;346:e7586

| | | Reporting Item | Page Number |
|---|---|---|---|
| **Administrative information** | | | |
| Title | #1 | Descriptive title identifying the study design, population, interventions, and, if applicable, trial acronym | 1 |
| Trial registration | #2a | Trial identifier and registry name. If not yet registered, name of intended registry | n/a |
| Trial registration: data set | #2b | All items from the World Health Organization Trial Registration Data Set | n/a |
| Protocol version | #3 | Date and version identifier | |
| Funding | #4 | Sources and types of financial, material, and other support | 2 |
| Roles and responsibilities: contributorship | #5a | Names, affiliations, and roles of protocol contributors | 17 |

For peer review only

| | | | |
|---|---|---|---|
| Roles and responsibilities: sponsor contact information | #5b | Name and contact information for the trial sponsor | n/a |
| Roles and responsibilities: sponsor and funder | #5c | Role of study sponsor and funders, if any, in study design; collection, management, analysis, and interpretation of data; writing of the report; and the decision to submit the report for publication, including whether they will have ultimate authority over any of these activities | n/a |
| Roles and responsibilities: committees | #5d | Composition, roles, and responsibilities of the coordinating centre, steering committee, endpoint adjudication committee, data management team, and other individuals or groups overseeing the trial, if applicable (see Item 21a for data monitoring committee) | n/a |
| **Introduction** | | | |
| Background and rationale | #6a | Description of research question and justification for undertaking the trial, including summary of relevant studies (published and unpublished) examining benefits and harms for each intervention | 3 |
| Background and rationale: choice of comparators | #6b | Explanation for choice of comparators | n/a |
| Objectives | #7 | Specific objectives or hypotheses | 4 |
| Trial design | #8 | Description of trial design including type of trial (eg, parallel group, crossover, factorial, single group), allocation ratio, and framework (eg, superiority, equivalence, non-inferiority, exploratory) | 4 |
| **Methods: Participants, interventions, and outcomes** | | | |
| Study setting | #9 | Description of study settings (eg, community clinic, academic hospital) and list of countries where data will be collected. Reference to where list of study sites can be obtained | 5 |

For peer review only

| | | | |
|---|---|---|---|
| Eligibility criteria | #10 | Inclusion and exclusion criteria for participants. If applicable, eligibility criteria for study centres and individuals who will perform the interventions (eg, surgeons, psychotherapists) | 7 |
| Interventions: description | #11a | Interventions for each group with sufficient detail to allow replication, including how and when they will be administered | 7-11 |
| Interventions: modifications | #11b | Criteria for discontinuing or modifying allocated interventions for a given trial participant (eg, drug dose change in response to harms, participant request, or improving / worsening disease) | n/a |
| Interventions: adherance | #11c | Strategies to improve adherence to intervention protocols, and any procedures for monitoring adherence (eg, drug tablet return; laboratory tests) | n/a |
| Interventions: concomitant care | #11d | Relevant concomitant care and interventions that are permitted or prohibited during the trial | n/a |
| Outcomes | #12 | Primary, secondary, and other outcomes, including the specific measurement variable (eg, systolic blood pressure), analysis metric (eg, change from baseline, final value, time to event), method of aggregation (eg, median, proportion), and time point for each outcome. Explanation of the clinical relevance of chosen efficacy and harm outcomes is strongly recommended | 9-10 |
| Participant timeline | #13 | Time schedule of enrolment, interventions (including any run-ins and washouts), assessments, and visits for participants. A schematic diagram is highly recommended (see Figure) | n/a |
| Sample size | #14 | Estimated number of participants needed to achieve study objectives and how it was determined, including clinical and statistical assumptions supporting any sample size calculations | n/a |
| Recruitment | #15 | Strategies for achieving adequate participant enrolment to reach target sample size | n/a |
| **Methods: Assignment of interventions (for controlled trials)** | | | |
| Allocation: sequence generation | #16a | Method of generating the allocation sequence (eg, computer-generated random numbers), and list of any factors for stratification. To reduce predictability of a random sequence, details of any planned restriction (eg, blocking) should be | n/a |

For peer review only

| | | | |
|---|---|---|---|
| | | provided in a separate document that is unavailable to those who enrol participants or assign interventions | |
| Allocation concealment mechanism | #16b | Mechanism of implementing the allocation sequence (eg, central telephone; sequentially numbered, opaque, sealed envelopes), describing any steps to conceal the sequence until interventions are assigned | n/a |
| Allocation: implementation | #16c | Who will generate the allocation sequence, who will enrol participants, and who will assign participants to interventions | n/a |
| Blinding (masking) | #17a | Who will be blinded after assignment to interventions (eg, trial participants, care providers, outcome assessors, data analysts), and how | n/a |
| Blinding (masking): emergency unblinding | #17b | If blinded, circumstances under which unblinding is permissible, and procedure for revealing a participant's allocated intervention during the trial | n/a |
| **Methods: Data collection, management, and analysis** | | | |
| Data collection plan | #18a | Plans for assessment and collection of outcome, baseline, and other trial data, including any related processes to promote data quality (eg, duplicate measurements, training of assessors) and a description of study instruments (eg, questionnaires, laboratory tests) along with their reliability and validity, if known. Reference to where data collection forms can be found, if not in the protocol | 7-10 |
| Data collection plan: retention | #18b | Plans to promote participant retention and complete follow-up, including list of any outcome data to be collected for participants who discontinue or deviate from intervention protocols | n/a |
| Data management | #19 | Plans for data entry, coding, security, and storage, including any related processes to promote data quality (eg, double data entry; range checks for data values). Reference to where details of data management procedures can be found, if not in the protocol | 9-10 |
| Statistics: outcomes | #20a | Statistical methods for analysing primary and secondary outcomes. Reference to where other details of the statistical analysis plan can be found, if not in the protocol | 9-10, 12 |

For peer review only

| | | | |
|---|---|---|---|
| Statistics: additional analyses | #20b | Methods for any additional analyses (eg, subgroup and adjusted analyses) | n/a |
| Statistics: analysis population and missing data | #20c | Definition of analysis population relating to protocol non-adherence (eg, as randomised analysis), and any statistical methods to handle missing data (eg, multiple imputation) | n/a |
| **Methods: Monitoring** | | | |
| Data monitoring: formal committee | #21a | Composition of data monitoring committee (DMC); summary of its role and reporting structure; statement of whether it is independent from the sponsor and competing interests; and reference to where further details about its charter can be found, if not in the protocol. Alternatively, an explanation of why a DMC is not needed | n/a |
| Data monitoring: interim analysis | #21b | Description of any interim analyses and stopping guidelines, including who will have access to these interim results and make the final decision to terminate the trial | n/a |
| Harms | #22 | Plans for collecting, assessing, reporting, and managing solicited and spontaneously reported adverse events and other unintended effects of trial interventions or trial conduct | n/a |
| Auditing | #23 | Frequency and procedures for auditing trial conduct, if any, and whether the process will be independent from investigators and the sponsor | n/a |
| **Ethics and dissemination** | | | |
| Research ethics approval | #24 | Plans for seeking research ethics committee / institutional review board (REC / IRB) approval | 2 |
| Protocol amendments | #25 | Plans for communicating important protocol modifications (eg, changes to eligibility criteria, outcomes, analyses) to relevant parties (eg, investigators, REC / IRBs, trial participants, trial registries, journals, regulators) | 2 |
| Consent or assent | #26a | Who will obtain informed consent or assent from potential trial participants or authorised surrogates, and how (see Item 32) | 8 |

For peer review only

| | | | |
|---|---|---|---|
| Consent or assent: ancillary studies | #26b | Additional consent provisions for collection and use of participant data and biological specimens in ancillary studies, if applicable | n/a |
| Confidentiality | #27 | How personal information about potential and enrolled participants will be collected, shared, and maintained in order to protect confidentiality before, during, and after the trial | n/a |
| Declaration of interests | #28 | Financial and other competing interests for principal investigators for the overall trial and each study site | 17 |
| Data access | #29 | Statement of who will have access to the final trial dataset, and disclosure of contractual agreements that limit such access for investigators | |
| Ancillary and post trial care | #30 | Provisions, if any, for ancillary and post-trial care, and for compensation to those who suffer harm from trial participation | n/a |
| Dissemination policy: trial results | #31a | Plans for investigators and sponsor to communicate trial results to participants, healthcare professionals, the public, and other relevant groups (eg, via publication, reporting in results databases, or other data sharing arrangements), including any publication restrictions | 2 |
| Dissemination policy: authorship | #31b | Authorship eligibility guidelines and any intended use of professional writers | n/a |
| Dissemination policy: reproducible research | #31c | Plans, if any, for granting public access to the full protocol, participant-level dataset, and statistical code | n/a |
| **Appendices** | | | |
| Informed consent materials | #32 | Model consent form and other related documentation given to participants and authorised surrogates | 18 |
| Biological specimens | #33 | Plans for collection, laboratory evaluation, and storage of biological specimens for genetic or molecular analysis in the current trial and for future use in ancillary studies, if applicable | n/a |

None The SPIRIT checklist is distributed under the terms of the Creative Commons Attribution License CC-BY-ND 3.0. This checklist can be completed online using https://www.goodreports.org/, a tool made by the EQUATOR Network in collaboration with Penelope.ai

For peer review only

BMJ Open

# Optimizing implementation strategies of the first scale-up of a primary care psychological intervention for common mental disorders in Sub-Saharan Africa: A Mixed Methods Study protocol for the Optimized Friendship Bench (OptFB)



| | |
|---|---|
| Journal: | *BMJ Open* |
| Manuscript ID | bmjopen-2020-045481.R2 |
| Article Type: | Protocol |
| Date Submitted by the Author: | 29-Jun-2021 |




| | |
|---|---|
| Complete List of Authors: | Verhey, Ruth ; University of Zimbabwe, Research Support Centre; Friendship Bench<br>Chitiyo, Charmaine; Friendship Bench Zimbabwe<br>Mboweni, Sandra; Friendship Bench Zimbabwe<br>Chiriseri, Ephraim; Friendship Bench Zimbabwe<br>Chibanda, Dixon; Friendship Bench Zimbabwe; University of Zimbabwe, Research Support Centre<br>Healey, Andy; King's College London, IOPPN<br>Wagenaar, Bradley; University of Washington, Department of epidemiology; University of Washington, Department of global health<br>Araya, Ricardo; Centre for Global Mental Health and Primary Care Research, |




| | |
|---|---|
| <b>Primary Subject Heading</b>: | Mental health |
| Secondary Subject Heading: | Global health, Communication |
| Keywords: | MENTAL HEALTH, PRIMARY CARE, PUBLIC HEALTH, QUALITATIVE RESEARCH |
| | |



SCHOLARONE™
Manuscripts

I, the Submitting Author has the right to grant and does grant on behalf of all authors of the Work (as defined in the below author licence), an exclusive licence and/or a non-exclusive licence for contributions from authors who are: i) UK Crown employees; ii) where BMJ has agreed a CC-BY licence shall apply, and/or iii) in accordance with the terms applicable for US Federal Government officers or employees acting as part of their official duties; on a worldwide, perpetual, irrevocable, royalty-free basis to BMJ Publishing Group Ltd ("BMJ") its licensees and where the relevant Journal is co-owned by BMJ to the co-owners of the Journal, to publish the Work in this journal and any other BMJ products and to exploit all rights, as set out in our licence.

The Submitting Author accepts and understands that any supply made under these terms is made by BMJ to the Submitting Author unless you are acting as an employee on behalf of your employer or a postgraduate student of an affiliated institution which is paying any applicable article publishing charge ("APC") for Open Access articles. Where the Submitting Author wishes to make the Work available on an Open Access basis (and intends to pay the relevant APC), the terms of reuse of such Open Access shall be governed by a Creative Commons licence – details of these licences and which Creative Commons licence will apply to this Work are set out in our licence referred to above.

Other than as permitted in any relevant BMJ Author's Self Archiving Policies, I confirm this Work has not been accepted for publication elsewhere, is not being considered for publication elsewhere and does not duplicate material already published. I confirm all authors consent to publication of this Work and authorise the granting of this licence.

For peer review only

# Optimizing implementation strategies of the first scale-up of a primary care psychological intervention for common mental disorders in Sub-Saharan Africa: A Mixed Methods Study protocol for the Optimized Friendship Bench (OptFB)

Author list:

Ruth Verhey [a,b]

Charmaine Chitiyo [a]

Sandra Mboweni [a]

Ephraim Chiriseri [a]

Dixon Chibanda [a,b,d]

Andy Healey [c]

Bradley H. Wagenaar [e,f]

Ricardo Araya [c]

[a] Friendship Bench Zimbabwe

[b] University of Zimbabwe

[c] King's College, London, UK

[d] London school of hygiene and tropical medicine, LSHTM, UK

[e] Department of Global Health, University of Washington, Settle, WA, USA

[f] Department of Epidemiology, University of Washington, Seattle, WA, USA

Abstract word count: 281

Body word count: 3650

Corresponding author:

Ruth Verhey

Address: 4 Weale Road, Milton Park Harare Zimbabwe

Tel: +263773857376 Email: ruth.verhey@friendshipbench.io



For peer review only - http://bmjopen.bmj.com/site/about/guidelines.xhtml

**Abstract**

Introduction: Common mental disorders (CMDs) are a leading cause of disability globally. CMDs are highly prevalent in Zimbabwe and have been addressed by an evidence-based, task-shifting psychological intervention called the Friendship Bench (FB). The task-shifted FB program guides clients through problem solving therapy. It was scaled-up across 36 implementation sites in Zimbabwe in 2016.

Methods and analysis: This study will employ a mixed-methods framework. It aims to: (1) Use quantitative survey methodologies organized around the RE-AIM evaluation framework to assess the current scale-up of the FB intervention and classify 36 clinics according to levels of performance; (2) Use qualitative focus group discussions and semi-structured interviews organized around the Consolidated Framework for Implementation Research (CFIR) to analyze determinants of implementation success, as well as elucidate heterogeneity in implementation strategies through comparing high- and low-performing clinics; and (3) Use the results from aims 1 and 2 to develop strategies to optimize the Friendship Bench intervention and apply this model in a cluster randomized controlled trial to evaluate potential improvements among low-performing clinics. The trial will be registered with the Pan African Clinical Trial Registry (www.pactr.org). The planned randomized controlled trial for the third research aim will be registered after completing aims one and two because the intervention is dependent on knowledge generated during these phases.

Ethics and dissemination: The research protocol received full authorization from the Medical Research Council of Zimbabwe (MRCZ A/242). It is anticipated that changes in data collection tools and consent forms will take place at all three phases of the study and approval from MRCZ will be sought. All interview partners will be asked for informed consent. The research team will prioritize open access publications to disseminate research results.



For peer review only - http://bmjopen.bmj.com/site/about/guidelines.xhtml

## Strengths and limitations of this study

- Few evidence-based psychological interventions offered at primary health care level have been successfully scaled-up in Sub-Saharan Africa; this study is designed to deliver detailed knowledge about factors that influence the scale-up of a primary care psychological intervention (the Friendship Bench) in an African setting.
- Two widely used implementation science models, RE-AIM and CFIR, will be used to evaluate the implementation of this intervention, which was scaled up in 2016.
- This study focuses on evaluating the scaling up of evidence-based interventions and developing and testing implementation strategies to potentially optimize the routine delivery of the Friendship Bench.
- A limitation is that comprehensive implementation data is only collected three years after the scale up exercise.

Key words: Friendship Bench, Optimization, common mental disorders, CFIR, RE-AIM, Low- and middle-income countries

For peer review only

## 1. Introduction:

In the past 10 years, it has become apparent that mental, neurological and substance use disorders (MNS) are among the leading causes of the global disease burden [1-3]. Research has shown that 4 out of every 10 people in low-and-middle-income countries (LMICs) suffer from mental disorders (de Boer et al. 2008, World Health Organization, 2009a) and evidence-based mental health interventions have become a focus of research and interest [4]. It has been observed that the poor are disproportionately affected by mental disorders [5 6]. Less than 5% of people living in some LMIC receive any adequate treatment for mental health disorders [7 8 9]. Particularly in low- and

middle-income countries (LMIC) the lack of resources, especially trained mental health professionals, causes sub-optimal detection and management of CMD [10-12]. Worldwide, efforts have been made to create sustainable and affordable mental health interventions in primary care [13-18]. In a recent systematic review, only four studies were detected that had evaluated the implementation of a depression intervention scaled-up in routine care [19]. As it stands, the benefit of these evidence-based interventions is not yet reaching those populations most at need across LMICs.

Zimbabwe, a country in Southern Africa with a population of 13 million has a large treatment gap for MNS. Studies show that over 30% of primary health care (PHC) users need mental health care services for mostly common mental disorders (CMD) and only 5% of these receive appropriate care [20]. Untreated CMD can also lead to worsening of clinical outcomes in chronic conditions such as HIV [21] and negatively affect economic outcomes too [5]. The Friendship Bench (FB) was developed in response to the existing treatment gap for mental health care in Zimbabwe and tested for its efficacy in a cluster randomized controlled trial (RCT) [22].

This task-shifted intervention is delivered by trained and supervised lay health workers (LHWs) who deliver problem solving therapy (PST) [23] on a bench located in primary health care clinics. In 2016, the FB intervention was scaled-up across Harare, Gweru and Chitungwiza and surrounding peri-urban communities in collaboration with the respective City Health departments [24]. The FB program was established in 72 City Health PHC clinics that are established in 36 sites (different clinic types can be found in the same site). This scaling-up exercise involved the training of more than 300 LHWs in the 3 cities in Zimbabwe [24]. Maintenance funding for FB activities is provided by the City Health department.

All lay health workers (LHWs) working for the FB PHC clinics in Harare, Gweru and Chitungwiza received the standard manualized training and supervision. While existing scientific evidence has shown that under ideal randomized trial conditions the FB intervention leads to clinically-significant reductions in symptoms, little implementation research has been carried out regarding the performance of Friendship Bench under routine conditions as the model is being further scaled-up across Zimbabwe.



For peer review only - http://bmjopen.bmj.com/site/about/guidelines.xhtml

This study will be of interest to implementation scientists, policymakers, and researchers working to scale-up primary care psychological interventions in low- and middle-income countries (LMICs) globally. Results from this study have the potential to inform future scale-up and maintenance of task-shared psychological interventions into routine Ministry of Health primary care settings.

Preliminary observations

Preliminary work had revealed that FB activities were irregular over the implementation sites. FB related data collection was often unreliable due to various reasons such as the delivering agents not having been trained on data collection, and the FB program data not being reported to the authorities as part of the clinic activities. Only estimates for client numbers for 2016-2018 with a program reach decline from 27,967 clients in 2016 to 6,688 in 2018 for all of the 36 sites were available. Sites in Harare had continued to offer the program. In the two other cities (Gweru and Chitungwiza) the health authorities had ceased to support the FB program and delivering agents had been told to focus on other programs such as HIV related activities. It was unclear how many FB activities had been carried out. In order to receive continued support, the FB program should be integrated with other PHC programs such as HIV care. Data collection efforts need to be simplified and delivering agents trained. Data needs to be gathered and analysed regularly using implementation science principles. Furthermore, the FB organization should engage closely with health care providers and policy makers to ensure successful and continued program implementation.

## 2. Overall Study Goal

This research uses a mixed-methods study design and widely-used implementation frameworks to systematically analyze the performance of clinics, determinants of this performance, including implementation strategies that might differentiate high- versus low-performing clinics, and develop and test an enhanced implementation strategy to improve the performance of clinics in three cities in Zimbabwe. The study is designed to be conducted in three phases with corresponding aims.

Firstly (aim 1), we plan to examine how the FB is performing under real-world implementation conditions and classify existing clinics with FB into high- versus low-performing sites using differences in RE-AIM outcomes [25 26].



For peer review only - http://bmjopen.bmj.com/site/about/guidelines.xhtml

Secondly (aim 2), we will analyse the determinants of heterogeneity in the results of phase 1 comparing high- versus low-performing clinics, mainly using the CFIR framework [27] and rigorously documenting changes to the original FB protocol and current implementation strategies in use.

Thirdly (aim 3), we will develop and test an optimized package of FB implementation strategies based on the results of phase 2 and measure the improvement among low performing clinics using RE-AIM outcomes.

### 3. Study setting:

The study will be conducted in primary health care clinics (PHC) in Harare, Gweru, and Chitungwiza.

Most of the clinics in the 3 cities are located in comparable areas which are characterized by high population density and informal income generating activities often occurring in the vicinity of the clinics. Depending on their size, PHC clinics serve between 20,000-80,000 people from the most socio-economically disadvantaged sectors of the population. Clinics are differentiated into poly, satellite, and family health service clinics according to the size of the clinic and the range of services offered.

The most comprehensive services are offered in a Polyclinic such as pre-, post- and perinatal care, opportunistic infections (for example TB treatment), and specialized NGO-based programs (HIV testing and management, male circumcision, communicable disease awareness). Satellite and Family health clinics (FHS) offer less services. Medical doctors are not permanently present but hold clinics on specific days in poly clinics. This influences the clinic user population's composition on these particular days (for example HIV clinic day).

Clinics in Harare, Chitungwiza and Gweru are grouped and located in the same geographical facility and these are counted as one Friendship Bench implementation site. Data will be collected in 36 implementation sites (n=28 in Harare; n=4 in Gweru; n=4 in Chitungwiza). Of these 26 Poly clinics, six are FHS and four satellite clinics (see figure 1).

Figure 1: Clinic type distribution for Harare, Gweru and Chitungwiza



For peer review only - http://bmjopen.bmj.com/site/about/guidelines.xhtml

Depending on their size and catchment area, FB implementation sites have between one (1) and fourteen (14) LHWs who deliver the FB intervention on benches in the clinic premises during clinic opening times. Clinic users are informed about the about FB services and mental health through group or individual talks in the clinic's waiting areas. Community members are also directly in contact with LHWs during outreach activities in the community.

## 4. Methods

This study proposes a rigorous analysis of the multiple interconnecting factors using two internationally recognized implementation research methods – the RE-AIM model [26] and the CFIR [27] which will be described in more detail below. Both conceptual frameworks have been used widely in implementation research for health care delivery in order to deepen the understanding and evaluation of interventions such as the Friendship Bench. The study has three research aims which are linked contextually to each other and are described in detail below.

Patient and Public Involvement

Patients and/or the public will be involved in the stakeholder meetings, they were not involved in the design, nor will they be involved in the study conduct, or reporting, or dissemination plans of this research project.

---

### 4.1 Methods Aim 1

A thorough analysis of the existing routine health information system data collected by the Harare, Gweru and Chitungwiza City Health authorities will be carried out to learn about the Friendship Bench activities at individual clinic level. This data consists of user numbers, age, gender, HIV status, clients' screening tool scores pre- and post-intervention as well as complete use of screening tool, and number of sessions.

We will use the RE-AIM evaluation framework to evaluate the current implementation performance of the FB intervention after three years of implementation experience. Routinely collected data will be used to assess the FB intervention's real-world and



For peer review only - http://bmjopen.bmj.com/site/about/guidelines.xhtml

pragmatic performance: Reach, Effectiveness, Adoption, Implementation, and Maintenance. The research team which consists of experienced global mental health researchers and clinicians will develop indicators for each of the RE-AIM domains using the www.re-aim.org website to support us and base our decisions on expert consensus and availability of data. These indicators will then be used to design a questionnaire to guide the RE-AIM related data collection. Each indicator will comprise a numerator and a denominator populated with data collected from the clinic records and the planned observations.

The data on the FB implementation will be analyzed for each of the 36 participating clinics. Routinely collected data includes clinical registries for both nurses and LHWs and data from the FB Register (commonly known as the “green book”) where the LHWs record beneficiary information.

In addition, LHWs will be observed during all aspects of their work, including giving health talks, interacting with clients, and delivering the FB intervention. We will observe and record whether all FB related tools such as questionnaires and intervention tools are used.

In order to collect additional necessary data for AIM 1, key respondents will be interviewed using a questionnaire that will be developed by the research team.

We plan to interview at least 2 LHWs per clinic and in clinics with more than 2 LHWs, we will interview 50% of the present LHWs by randomly selecting them. Papers with their names will be put in a container from which a RA will pull out the appropriate number in the LHWs' presence. We will always interview the supervisor LHW of each clinic if this position is taken in a particular clinic. We will also interview the nurse in charge in every clinic and the associated district health promoting officers (DHPOs) (n=10). Data will be collected from June to September 2019 in all participating sites.

The data collection will be carried out by two research coordinators who will lead two teams of four trained and supervised research assistants (RAs). The teams will visit each clinic for two days. The clinics will be sensitized about the FB team visit a week prior. The research assistants will be trained to interview, to observe and record the FB related activities in the clinic and how to enter the data digitally using tablet computers. They will be trained on data checking, cleaning and uploading.

For peer review only

Furthermore, we are planning to audio-record FB sessions with consenting clients (two per site, n=72). We will approach, where possible, all incoming clients seeking services and ask them for informed consent to allow us to record their session with the FB LHWs. We aim to record as many as possible but at least 2 per site.

The recordings will be translated, transcribed and rated according to the Friendship Bench fidelity checklist.

The FB fidelity checklist assesses for communication skills of the counselor, the level of psychoeducation that is done, and the adherence to the problem-solving therapy steps that the FB counselor is trained to deliver (see Supplementary Appendix A for full fidelity checklist which was developed for the RCT [22]). The assessments of audio recordings will be done by trained FB research team members who will prepare an audio-recorder which will be left with the FB counselor after a client has given consent. The audio recording device will be retrieved by the research assistant when the LHW has indicated that the session is done.

In the event that no clients come to the clinic on both days that the FB team visits the site or no client consents to have their session audio-recorded, this will be entered as missing. Due to logistic and financial constraints a repeat visit to a particular clinic will not be possible.

All respondents will be asked to answer the questions with regards to FB activities in the past month. According to their position with regards to FB activities, questions might be formulated slightly differently.

The questionnaires will be administered using tablet computers (Lenovo), all observational data will be entered digitally after their correctness has been ascertained by asking interviewees to show evidence as applicable. Questionnaires and observation guides are programmed into the tablets using Kobotoolbox (https://www.kobotoolbox.org) which is a data collection tool. Collected data will be cleaned and uploaded daily to a password secured server.

The research team will also observe FB specific activities such as health and 'mobilization' talks that are given by the clinic staff including the LHWs whilst patients are waiting to be seen.


For peer review only - http://bmjopen.bmj.com/site/about/guidelines.xhtml

A stakeholder meeting will be held once Aim 1 data is completed and the data is analyzed. At this meeting, the research team will present the results from Aim 1 and discuss potential reasons why we might see the differences in implementation across sites with stakeholders. This meeting will be attended by all relevant clinic staff, health authority officers as well as clients. Information from stakeholders will be used to select and prioritize CFIR constructs to include in qualitative interview guides for Aim 2.

**4.1.1 Data Analysis Aim 1**

The goal of Aim 1 is to classify the 36 FB implementation sites on their performance based on the RE-AIM outcomes. Our methods will follow similar classification efforts previously published [28]. Clinics will be first ranked according to their performance within each individual measure. Clinics score on all indicators within one construct (for example reach) will be averaged. For each of the RE-AIM constructs, every clinic will thus have an averaged ranking.

These domain-based rankings will be averaged per clinic rankings giving an overall ranking by calculating simple means of all domain rankings. This procedure will be carried out by two independent individuals and any differences will lead to a redoing of the process. In case of same outcomes for clinics, we will treat these particular clinics as being on the same rank. This will give us a final composite rank for each clinic which will be used to determine the 10 highest and 10 lowest performing clinics that will be qualitatively assessed in Aim 2.

**4.2 Methods Aim 2**

With the aim to understand the determinants of implementation success, as well as differences in implementation strategies employed, Aim 2 will utilize focus-group discussions organized around the Consolidated Framework for Implementation Research (CFIR) [29-31]. Through these qualitative methods, we aim to gain a deeper understanding of the factors that contribute to the successful implementation comparing high- with low-performing clinics. The CFIR framework focuses on an overview of potential multi-level determinants of health care delivery. It was designed to help understand integrated implementation determinants across multiple levels (clients; implementers; organizations; contexts; processes).


For peer review only - http://bmjopen.bmj.com/site/about/guidelines.xhtml

For the present study, we will focus on determinants of implementation success, taking lessons from both high- and low-performing clinics to inform the development of an improved package of implementation strategies targeting identified barriers.

Focus group discussions (FDGs) with key informants (LHWs, nurses, DHPOs, clients) of the 10 high and 10 low performing clinics will be carried out by trained qualitative researchers. The FB specific interview guides for these group discussions and interviews will be developed by the study team in a sequence of internal project meetings using the online technical support website www.cfirguide.org. The results of Aim 1 will guide us in designing the interview guides for the focus group discussions. The outcome of the stakeholders meeting in which we present the results of Aim 1 will also give us insight on the importance of constructs which we will take into account when designing the CFIR interview guides.

Interview guides will be translated into the local language Shona and all group discussions will be audio-recorded, transcribed and translated to English. All discussions will be held in the local language.

The FGD participants will be selected from all 10 low and high performing clinics, respectively. We will interview LHWs, nurses, DHPOs in their role as implementers as well as clients as recipients of the intervention. Nurses and DHPOs will be invited to joined meetings. We will conduct FGDs for all available LHWs at every selected clinic. We will ask the selected LHWs to purposively suggest 2 clients each, whom we will then invite to FGDs in each of the selected clinics. In case a client declines participation, we will ask for another suggestion.

Focus group discussions will take place in clinics or, if not possible, in the Friendship Bench office in Harare.

#### 4.2 1 Data Analysis Aim 2

CFIR analyses will follow the original Damschroder methodology previously published [30]. Briefly, two independent local Zimbabwean reviewers will code each FGD transcript according to the selected CFIR constructs. Differences will be discussed and revised until final codes are agreed on. Facility-level case memos will be organized by the relevant CFIR construct, using each new transcript to confirm and refine statements until all transcripts are coded. This process will be closely supported by the whole



For peer review only - http://bmjopen.bmj.com/site/about/guidelines.xhtml

research team. Each clinic will have two case memos, one for LHWs and other implementers and one for clients.

Using case memos and supporting transcripts, the same two coders will independently rate CFIR constructs on valence (X (mixed); 0 (neutral); + (construct has a positive effect on implementation) or – (construct has a negative influence on implementation). Once drafted, the entire research team will meet and use a deliberated consensus to finalize memos, constructs, and valence. These data will be mapped on a matrix template with the goal of identifying constructs that differ between facilities with high and low performance to identify factors relevant for the success of the implementation. Analyses will progress with visual inspection of patterns in constructs and valence by high versus low performing clinics, as well as examining median and mean valence by high versus low performing clinics. Once distinguishing constructs are identified, the team will re-review case memos and coded transcripts to gather more information on constructs.

**4.3 Aim 3**

In Aim 3, we will develop a package of optimized Friendship Bench (OptFB) implementation strategies matched to key barriers identified in the previous phases of this study. Using CFIR data on barriers / facilitators to high-quality FB implementation, we will use the CFIR-ERIC matching tool to examine and select implementation strategies to address key CFIR constructs discriminating between high and low performing clinics in Aim 1 (https://cfirguide.org/choosing-strategies/) [32 33]. Once a preliminary list is developed by our team, the CFIR-Expert Recommendation for Implementation Change (ERIC) matching tool [32] will be used to prioritize those strategies that are found to be most likely to address CFIR barriers in low-performing clinics [33 34].

We will engage in a participatory stakeholder Delphi rating exercise to select specific strategies. This will be followed by the research team specifying and tailoring the strategies for the Zimbabwean context by including the additional information gained from the stakeholders. Aspects of feasibility, affordability and effectiveness will guide this process in order for the package to be meaningful and effective [35]. Strategies currently in use by high performing clinics will be also considered for the optimized Friendship Bench (OptFB) implementation strategies.



For peer review only - http://bmjopen.bmj.com/site/about/guidelines.xhtml

This OptFB package or intervention of improved strategies will be tested in low-performing clinics. Ongoing RE-AIM data is being collected on a monthly basis in each clinic. Using these data on RE-AIM outcomes, we will re-classify clinics using a similar process as in Aim 1. We will then identify the 18 lowest performing clinics and randomly select 12 clinics to deliver the OptFB and 6 to act as control clinics over a period of 6 months. The primary outcome will be a composite measure of RE-AIM indicators (Reach, Effectiveness, Adoption and Implementation and Maintenance) estimated at 6-month after the commencement of the implementation of the OptFB intervention. We will estimate changes in this composite measure of implementation before and at 6 months after starting the delivery of OptFB in all clinics. We will compare the difference in means or proportions between the clinics receiving the OptFB and the control clinics using the routinely collected data. Secondary outcomes will examine performance of each of the RE-AIM outcomes separately and clinical effectiveness results at individual level. The latter will be based on individual scores ~~to~~ on the SSQ on a minimum of 20 random individuals per clinic during the 6-month period.

No sample size calculation has been estimated since there are no previous studies on which to estimate an effect size, the number of clinics is small, and the main outcomes are averaged data representing clusters. Nonetheless, we expect to see larger improvements in the RE-AIM composite index score in the clinics receiving OptFB compared to the control clinics over the 6 months. As a secondary outcome measure, clinical effectiveness will be assessed based on changes on SSQ scores from baseline to 6 months for a sample of 360 individuals (18 clinics with 20 individuals each), but we do not expect this sample would have enough power to detect small differences in effectiveness across the two group of clinics. Thus, comparisons on clinical effectiveness must be considered purely descriptive and exploratory and interpreted with caution. In any case, the main outcomes of interest in this study are implementation outcomes subsumed under the domains included in the RE-AIM framework.

### 4.3.1 Data analysis Aim 3

We will use a difference-in-differences analysis comparing the groups over time. Means or proportions on outcome data will be compared across groups using descriptive statistics. Regression models will be used to estimate the effect of the



For peer review only - http://bmjopen.bmj.com/site/about/guidelines.xhtml

intervention on the main outcomes. General estimating equations with robust standard errors will be used to control for clustering. Potential confounders will be determined a priori and included in the regression models. Standard errors, confidence intervals, and p-values will be obtained. A similar secondary analysis will be conducted with the secondary outcome measures.

### 4.4 Health economic analysis

Site-level data will be collected on fidelity to the OptFB implementation strategies, along with activities and resource inputs required to deliver improvement strategies and OptFB delivery costs. Economic modelling will be used to combine this information with data and evidence on clinical impact and implementation effectiveness to evaluate the cost-effectiveness of the OptFB program [36].

We will also revisit clinics and re-engage with stakeholders in ~~into~~ FGD to explore level of change in the identified CFIR domains in the intervention arm clinics.

After completion of the trial, the strategy will also be implemented in the control arm clinics to increase the overall performance in all of participating lower performing clinics.

## 5. Discussion

This study will contribute to the knowledge about scaling up of an evidence-based task-shifted intervention in a LMIC. This is a unique opportunity to analyze the Friendship Bench in a real-world setting. As mentioned above, not many interventions have been scaled up from LMICs and therefore there is a dearth of information on how implementation strategies can be used in order to ensure a strong scaling up. With this study we hope to learn which barriers and enablers are at play in the FB scale up process. This is particularly important for us as we are expanding the FB services throughout Zimbabwe and beyond to meet the population's needs for accessible and acceptable mental health care. This effort has to be undertaken with the aim of having high fidelity to the program while considering contextual aspects. Using implementation science principles will help us to give theoretical justification and describe specifications for application for those implementation strategies that we will devise after having gone through the different

For peer review only

stages of this research process. Evidence-based, clear and applicable guidelines of how to implement our evidence-based intervention in primary health care settings will be created and can then subsequently be used to ensure a strong implementation of FB.

## 6. Ethics and dissemination

This research protocol has been approved by the Medical Research Council Zimbabwe (MRCZ), MRCZ/A/2428 and the Joint Research Council (JREC), 79/19. Results will be disseminated in peer-reviewed journals and conferences.

## References

1. Vigo D, Thornicroft G, Atun R. Estimating the true global burden of mental illness. *Lancet Psychiatry* 2016;3(2):171-8. doi: 10.1016/S2215-0366(15)00505-2 [published Online First: 2016/02/07]
2. Kessler RC, Aguilar-Gaxiola S, Alonso J, et al. The global burden of mental disorders: an update from the WHO World Mental Health (WMH) surveys. *Epidemiol Psichiatr Soc* 2009;18(1):23-33. doi: 10.1017/s1121189x00001421 [published Online First: 2009/04/22]
3. Whiteford HA, Ferrari AJ, Degenhardt L, et al. The global burden of mental, neurological and substance use disorders: an analysis from the Global Burden of Disease Study 2010. *PLoS One* 2015;10(2):e0116820. doi: 10.1371/journal.pone.0116820 [published Online First: 2015/02/07]
4. Steel Z, Marnane C, Iranpour C, et al. The global prevalence of common mental disorders: a systematic review and meta-analysis 1980–2013. *International journal of epidemiology* 2014:dyu038.
5. Lund C, De Silva M, Plagerson S, et al. Poverty and mental disorders: breaking the cycle in low-income and middle-income countries. *The Lancet* 2011;378(9801):1502-14.
6. Evans-Lacko S, Aguilar-Gaxiola S, Al-Hamzawi A, et al. Socio-economic variations in the mental health treatment gap for people with anxiety, mood, and substance use disorders: results from the WHO World Mental Health (WMH) surveys. *Psychological medicine* 2018;48(9):1560-71. doi: 10.1017/S0033291717003336 [published Online First: 2017/11/28]
7. Wang PS, Aguilar-Gaxiola S, Alonso J, et al. Use of mental health services for anxiety, mood, and substance disorders in 17 countries in the WHO world mental health surveys. *The Lancet* 2007;370(9590):841-50.



For peer review only - http://bmjopen.bmj.com/site/about/guidelines.xhtml

8. Mojtabai R, Olfson M, Sampson NA, et al. Barriers to mental health treatment: results from the National Comorbidity Survey Replication. *Psychological medicine* 2011;41(8):1751-61. doi: 10.1017/S0033291710002291 [published Online First: 2010/12/08]

9. Kohn R, Saxena S, Levav I, et al. The treatment gap in mental health care. *Bull World Health Organ* 2004;82(11):858-66. doi: /S0042-96862004001100011 [published Online First: 2005/01/11]

10. Prince M, Patel V, Saxena S, et al. No health without mental health. *The lancet* 2007;370(9590):859-77.

11. Alonso J, Liu Z, Evans-Lacko S, et al. Treatment gap for anxiety disorders is global: Results of the World Mental Health Surveys in 21 countries. *Depress Anxiety* 2018;35(3):195-208. doi: 10.1002/da.22711 [published Online First: 2018/01/23]

12. Wang PS, Angermeyer M, Borges G, et al. Delay and failure in treatment seeking after first onset of mental disorders in the World Health Organization's World Mental Health Survey Initiative. *World Psychiatry* 2007;6(3):177-85. [published Online First: 2008/01/12]

13. Araya R, Rojas G, Fritsch R, et al. Treating depression in primary care in low-income women in Santiago, Chile: a randomised controlled trial. *Lancet* 2003;361(9362):995-1000. doi: 10.1016/S0140-6736(03)12825-5

14. Patel V, Thornicroft G. Packages of care for mental, neurological, and substance use disorders in low-and middle-income countries: PLoS Medicine Series. *PLoS medicine* 2009;6(10):e1000160.

15. Chatterjee S, Chowdhary N, Pednekar S, et al. Integrating evidence-based treatments for common mental disorders in routine primary care: feasibility and acceptability of the MANAS intervention in Goa, India. *World Psychiatry* 2008;7(1):39-46.

16. Rahman A, Malik A, Sikander S, et al. Cognitive behaviour therapy-based intervention by community health workers for mothers with depression and their infants in rural Pakistan: a cluster-randomised controlled trial. *The Lancet* 2008;372(9642):902-09.

17. Bolton P, Bass J, Neugebauer R, et al. Group interpersonal psychotherapy for depression in rural Uganda: a randomized controlled trial. *Jama* 2003;289(23):3117-24.

18. Lund C, Alem A, Schneider M, et al. Generating evidence to narrow the treatment gap for mental disorders in sub-Saharan Africa: rationale, overview and methods of AFFIRM. *Epidemiol Psychiatr Sci* 2015;24(3):233-40. doi: 10.1017/S2045796015000281 [published Online First: 2015/04/03]

19. Wagenaar BH, Hammett, W.H., Jackson, C., Atkins, D.L., Belus, J.M. and Kemp, C.G. Implementation outcomes and strategies for depression interventions in low-and middle-income countries: a systematic review. . *Global Mental Health* 2020;7

20. Chibanda D, Mesu P, Kajawu L, et al. Problem-solving therapy for depression and common mental disorders in Zimbabwe: piloting a task-shifting primary mental health care intervention in a population with a high prevalence of people living with HIV. *BMC public health* 2011;11:828. doi: 10.1186/1471-2458-11-828 [published Online First: 2011/10/28]

21. Antelman G, Kaaya S, Wei R, et al. Depressive symptoms increase risk of HIV disease progression and mortality among women in Tanzania. *Journal of acquired immune deficiency syndromes* 2007;44(4):470-7. doi: 10.1097/QAI.0b013e31802f1318

22. Chibanda D, Weiss HA, Verhey R, et al. Effect of a Primary Care-Based Psychological Intervention on Symptoms of Common Mental Disorders in Zimbabwe: A



For peer review only - http://bmjopen.bmj.com/site/about/guidelines.xhtml

Randomized Clinical Trial. *JAMA* 2016;316(24):2618-26. doi: 10.1001/jama.2016.19102 [published Online First: 2016/12/28]

23. Chibanda D, Bowers T, Verhey R, et al. The Friendship Bench programme: a cluster randomised controlled trial of a brief psychological intervention for common mental disorders delivered by lay health workers in Zimbabwe. *Int J Ment Health Syst* 2015;9:21. doi: 10.1186/s13033-015-0013-y [published Online First: 2015/01/01]
24. Chibanda D, Verhey R, Munetsi E, et al. Scaling up interventions for depression in sub-Saharan Africa: lessons from Zimbabwe. *Glob Ment Health (Camb)* 2016;3:e13. doi: 10.1017/gmh.2016.8 [published Online First: 2016/04/11]
25. Glasgow RE, Vogt TM, Boles SM. Evaluating the public health impact of health promotion interventions: the RE-AIM framework. *Am J Public Health* 1999;89(9):1322-27.
26. Glasgow RE, Vogt TM, Boles SM. Evaluating the public health impact of health promotion interventions: the RE-AIM framework. *American journal of public health* 1999;89(9):1322-7. doi: 10.2105/ajph.89.9.1322 [published Online First: 1999/09/04]
27. Damschroder LJ, Aron DC, Keith RE, et al. Fostering implementation of health services research findings into practice: a consolidated framework for advancing implementation science. *Implement Sci* 2009;4:50. doi: 10.1186/1748-5908-4-50 [published Online First: 2009/08/12]
28. Farris RP, Will JC, Khavjou O, et al. Beyond effectiveness: evaluating the public health impact of the WISEWOMAN program. *American journal of public health* 2007;97(4):641-47.
29. Breimaier HE, Heckemann B, Halfens RJ, et al. The Consolidated Framework for Implementation Research (CFIR): a useful theoretical framework for guiding and evaluating a guideline implementation process in a hospital-based nursing practice. *BMC Nurs* 2015;14:43. doi: 10.1186/s12912-015-0088-4 [published Online First: 2015/08/14]
30. Damschroder LJ, Lowery JC. Evaluation of a large-scale weight management program using the consolidated framework for implementation research (CFIR). *Implement Sci* 2013;8:51. doi: 10.1186/1748-5908-8-51 [published Online First: 2013/05/15]
31. Damschroder LJ, Aron DC, Keith RE, et al. Fostering implementation of health services research findings into practice: a consolidated framework for advancing implementation science. *Implementation science* 2009;4(1):50.
32. Powell BJ, Waltz TJ, Chinman MJ, et al. A refined compilation of implementation strategies: results from the Expert Recommendations for Implementing Change (ERIC) project. *Implement Sci* 2015;10:21. doi: 10.1186/s13012-015-0209-1 [published Online First: 2015/04/19]
33. Waltz TJ, Powell BJ, Fernandez ME, et al. Choosing implementation strategies to address contextual barriers: diversity in recommendations and future directions. *Implement Sci* 2019;14(1):42. doi: 10.1186/s13012-019-0892-4 [published Online First: 2019/05/01]
34. Godbee K, Gunn J, Lautenschlager NT, et al. Refined conceptual model for implementing dementia risk reduction: incorporating perspectives from Australian general practice. *Aust J Prim Health* 2020;26(3):247-55. doi: 10.1071/PY19249 [published Online First: 2020/05/28]

For peer review only

35. Cooper LA, Hill MN, Powe NR. Designing and evaluating interventions to eliminate racial and ethnic disparities in health care. *J Gen Intern Med* 2002;17(6):477-86. doi: 10.1046/j.1525-1497.2002.10633.x [published Online First: 2002/07/23]
36. Briggs A, Sculpher, M., & Claxton, K. . Decision modelling for health economic evaluation. Oxford: Oup Oxford 2006.

## Footnotes

### Contributors

RA designed the study and received the grant. RA is the overall PI. RV and DC are leading the study locally. CC will have oversight as project coordinator together with SM as her assistant over the data collection in all phases. BW is leading on the implantation science aspects. EC is conducting data cleaning and assisting with analysis. AH is leading on the health economics analysis. All authors will contribute to the development of questionnaires, interview guides and the strategies for the intervention. RV wrote the first version of the protocol paper. All authors contributed by critically reviewing all further drafts and approving of the final paper.

### Funding

This work was supported by Global Alliance for Chronic Diseases (GACD) through the Medical Research Council Grant number: MRC UKRI MR/S004270/1.

**Competing interests** None declared.

**Patient consent for publication** Not required.



For peer review only - http://bmjopen.bmj.com/site/about/guidelines.xhtml

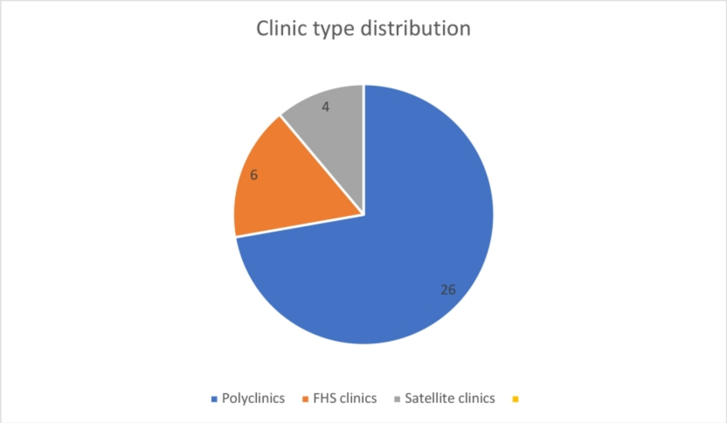


Figure 1: Clinic type distribution for Harare, Gweru and Chitungwiza

## Table 1. Checklist for audio-recorded sessions

## LHW FIDELITY CHECKLIST

**LHW Name**__________________________ **Client's Name**__________________________
**Date** / / **Site Code** ☐☐

| | | | |
|---|---|---|---|
| ***(Tick where appropriate)*** | | **YES** | **NO** |
| 1. | LHW introduced self to client and asked client to introduce self | ☐ | ☐ |
| 2. | Psycho-education done properly | | |
| | a. Linked HIV to kufungisisa | ☐ | ☐ |
| | b. Adherence | ☐ | ☐ |
| | c. Diet advise done | ☐ | ☐ |
| 3. | Problems presented by client | ☐ | ☐ |
| 4. | LHW listening and acknowledging | ☐ | ☐ |
| 5. | LHW gives summary of problems | ☐ | ☐ |
| 6. | Client selected problem, not LHW | ☐ | ☐ |
| 7. | LHW and client discuss problem identified by client | ☐ | ☐ |
| 8. | Client identifies solutions to the problem identified | ☐ | ☐ |
| 9. | LHW and client identify task for client to work with | ☐ | ☐ |
| 10. | Session closure and next correct review date | ☐ | ☐ |
| | ***Total*** | ____/10 | ___/10 |

# Reporting checklist for protocol of a clinical trial.

Based on the SPIRIT guidelines.

## Instructions to authors

Complete this checklist by entering the page numbers from your manuscript where readers will find each of the items listed below.

Your article may not currently address all the items on the checklist. Please modify your text to include the missing information. If you are certain that an item does not apply, please write "n/a" and provide a short explanation.

Upload your completed checklist as an extra file when you submit to a journal.

In your methods section, say that you used the SPIRITreporting guidelines, and cite them as:

Chan A-W, Tetzlaff JM, Gøtzsche PC, Altman DG, Mann H, Berlin J, Dickersin K, Hróbjartsson A, Schulz KF, Parulekar WR, Krleža-Jerić K, Laupacis A, Moher D. SPIRIT 2013 Explanation and Elaboration: Guidance for protocols of clinical trials. BMJ. 2013;346:e7586

| | | Reporting Item | Page Number |
|---|---|---|---|
| **Administrative information** | | | |
| Title | #1 | Descriptive title identifying the study design, population, interventions, and, if applicable, trial acronym | 1 |
| Trial registration | #2a | Trial identifier and registry name. If not yet registered, name of intended registry | n/a |
| Trial registration: data set | #2b | All items from the World Health Organization Trial Registration Data Set | n/a |
| Protocol version | #3 | Date and version identifier | |
| Funding | #4 | Sources and types of financial, material, and other support | 2 |
| Roles and responsibilities: contributorship | #5a | Names, affiliations, and roles of protocol contributors | 17 |

For peer review only

| | | | |
|---|---|---|---|
| Roles and responsibilities: sponsor contact information | #5b | Name and contact information for the trial sponsor | n/a |
| Roles and responsibilities: sponsor and funder | #5c | Role of study sponsor and funders, if any, in study design; collection, management, analysis, and interpretation of data; writing of the report; and the decision to submit the report for publication, including whether they will have ultimate authority over any of these activities | n/a |
| Roles and responsibilities: committees | #5d | Composition, roles, and responsibilities of the coordinating centre, steering committee, endpoint adjudication committee, data management team, and other individuals or groups overseeing the trial, if applicable (see Item 21a for data monitoring committee) | n/a |
| **Introduction** | | | |
| Background and rationale | #6a | Description of research question and justification for undertaking the trial, including summary of relevant studies (published and unpublished) examining benefits and harms for each intervention | 3 |
| Background and rationale: choice of comparators | #6b | Explanation for choice of comparators | n/a |
| Objectives | #7 | Specific objectives or hypotheses | 4 |
| Trial design | #8 | Description of trial design including type of trial (eg, parallel group, crossover, factorial, single group), allocation ratio, and framework (eg, superiority, equivalence, non-inferiority, exploratory) | 4 |
| **Methods: Participants, interventions, and outcomes** | | | |
| Study setting | #9 | Description of study settings (eg, community clinic, academic hospital) and list of countries where data will be collected. Reference to where list of study sites can be obtained | 5 |

For peer review only

| | | | |
|---|---|---|---|
| Eligibility criteria | #10 | Inclusion and exclusion criteria for participants. If applicable, eligibility criteria for study centres and individuals who will perform the interventions (eg, surgeons, psychotherapists) | 7 |
| Interventions: description | #11a | Interventions for each group with sufficient detail to allow replication, including how and when they will be administered | 7-11 |
| Interventions: modifications | #11b | Criteria for discontinuing or modifying allocated interventions for a given trial participant (eg, drug dose change in response to harms, participant request, or improving / worsening disease) | n/a |
| Interventions: adherance | #11c | Strategies to improve adherence to intervention protocols, and any procedures for monitoring adherence (eg, drug tablet return; laboratory tests) | n/a |
| Interventions: concomitant care | #11d | Relevant concomitant care and interventions that are permitted or prohibited during the trial | n/a |
| Outcomes | #12 | Primary, secondary, and other outcomes, including the specific measurement variable (eg, systolic blood pressure), analysis metric (eg, change from baseline, final value, time to event), method of aggregation (eg, median, proportion), and time point for each outcome. Explanation of the clinical relevance of chosen efficacy and harm outcomes is strongly recommended | 9-10 |
| Participant timeline | #13 | Time schedule of enrolment, interventions (including any run-ins and washouts), assessments, and visits for participants. A schematic diagram is highly recommended (see Figure) | n/a |
| Sample size | #14 | Estimated number of participants needed to achieve study objectives and how it was determined, including clinical and statistical assumptions supporting any sample size calculations | n/a |
| Recruitment | #15 | Strategies for achieving adequate participant enrolment to reach target sample size | n/a |
| **Methods: Assignment of interventions (for controlled trials)** | | | |
| Allocation: sequence generation | #16a | Method of generating the allocation sequence (eg, computer-generated random numbers), and list of any factors for stratification. To reduce predictability of a random sequence, details of any planned restriction (eg, blocking) should be | n/a |

For peer review only

| | | | |
|---|---|---|---|
| | | provided in a separate document that is unavailable to those who enrol participants or assign interventions | |
| Allocation concealment mechanism | #16b | Mechanism of implementing the allocation sequence (eg, central telephone; sequentially numbered, opaque, sealed envelopes), describing any steps to conceal the sequence until interventions are assigned | n/a |
| Allocation: implementation | #16c | Who will generate the allocation sequence, who will enrol participants, and who will assign participants to interventions | n/a |
| Blinding (masking) | #17a | Who will be blinded after assignment to interventions (eg, trial participants, care providers, outcome assessors, data analysts), and how | n/a |
| Blinding (masking): emergency unblinding | #17b | If blinded, circumstances under which unblinding is permissible, and procedure for revealing a participant's allocated intervention during the trial | n/a |
| **Methods: Data collection, management, and analysis** | | | |
| Data collection plan | #18a | Plans for assessment and collection of outcome, baseline, and other trial data, including any related processes to promote data quality (eg, duplicate measurements, training of assessors) and a description of study instruments (eg, questionnaires, laboratory tests) along with their reliability and validity, if known. Reference to where data collection forms can be found, if not in the protocol | 7-10 |
| Data collection plan: retention | #18b | Plans to promote participant retention and complete follow-up, including list of any outcome data to be collected for participants who discontinue or deviate from intervention protocols | n/a |
| Data management | #19 | Plans for data entry, coding, security, and storage, including any related processes to promote data quality (eg, double data entry; range checks for data values). Reference to where details of data management procedures can be found, if not in the protocol | 9-10 |
| Statistics: outcomes | #20a | Statistical methods for analysing primary and secondary outcomes. Reference to where other details of the statistical analysis plan can be found, if not in the protocol | 9-10, 12 |

For peer review only

| | | | |
|---|---|---|---|
| Statistics: additional analyses | #20b | Methods for any additional analyses (eg, subgroup and adjusted analyses) | n/a |
| Statistics: analysis population and missing data | #20c | Definition of analysis population relating to protocol non-adherence (eg, as randomised analysis), and any statistical methods to handle missing data (eg, multiple imputation) | n/a |
| **Methods: Monitoring** | | | |
| Data monitoring: formal committee | #21a | Composition of data monitoring committee (DMC); summary of its role and reporting structure; statement of whether it is independent from the sponsor and competing interests; and reference to where further details about its charter can be found, if not in the protocol. Alternatively, an explanation of why a DMC is not needed | n/a |
| Data monitoring: interim analysis | #21b | Description of any interim analyses and stopping guidelines, including who will have access to these interim results and make the final decision to terminate the trial | n/a |
| Harms | #22 | Plans for collecting, assessing, reporting, and managing solicited and spontaneously reported adverse events and other unintended effects of trial interventions or trial conduct | n/a |
| Auditing | #23 | Frequency and procedures for auditing trial conduct, if any, and whether the process will be independent from investigators and the sponsor | n/a |
| **Ethics and dissemination** | | | |
| Research ethics approval | #24 | Plans for seeking research ethics committee / institutional review board (REC / IRB) approval | 2 |
| Protocol amendments | #25 | Plans for communicating important protocol modifications (eg, changes to eligibility criteria, outcomes, analyses) to relevant parties (eg, investigators, REC / IRBs, trial participants, trial registries, journals, regulators) | 2 |
| Consent or assent | #26a | Who will obtain informed consent or assent from potential trial participants or authorised surrogates, and how (see Item 32) | 8 |

For peer review only

| | | | |
|---|---|---|---|
| Consent or assent: ancillary studies | #26b | Additional consent provisions for collection and use of participant data and biological specimens in ancillary studies, if applicable | n/a |
| Confidentiality | #27 | How personal information about potential and enrolled participants will be collected, shared, and maintained in order to protect confidentiality before, during, and after the trial | n/a |
| Declaration of interests | #28 | Financial and other competing interests for principal investigators for the overall trial and each study site | 17 |
| Data access | #29 | Statement of who will have access to the final trial dataset, and disclosure of contractual agreements that limit such access for investigators | |
| Ancillary and post trial care | #30 | Provisions, if any, for ancillary and post-trial care, and for compensation to those who suffer harm from trial participation | n/a |
| Dissemination policy: trial results | #31a | Plans for investigators and sponsor to communicate trial results to participants, healthcare professionals, the public, and other relevant groups (eg, via publication, reporting in results databases, or other data sharing arrangements), including any publication restrictions | 2 |
| Dissemination policy: authorship | #31b | Authorship eligibility guidelines and any intended use of professional writers | n/a |
| Dissemination policy: reproducible research | #31c | Plans, if any, for granting public access to the full protocol, participant-level dataset, and statistical code | n/a |
| **Appendices** | | | |
| Informed consent materials | #32 | Model consent form and other related documentation given to participants and authorised surrogates | 18 |
| Biological specimens | #33 | Plans for collection, laboratory evaluation, and storage of biological specimens for genetic or molecular analysis in the current trial and for future use in ancillary studies, if applicable | n/a |

None The SPIRIT checklist is distributed under the terms of the Creative Commons Attribution License CC-BY-ND 3.0. This checklist can be completed online using https://www.goodreports.org/, a tool made by the EQUATOR Network in collaboration with Penelope.ai

For peer review only
